# Testing effects of bottom‐up factors, grazing, and competition on New Zealand rocky intertidal algal communities

**DOI:** 10.1002/ece3.10704

**Published:** 2024-03-07

**Authors:** Barbara J. Spiecker, Bruce A. Menge

**Affiliations:** ^1^ Department of Integrative Biology Oregon State University Corvallis Oregon USA; ^2^ Department of Biological Sciences University of New Hampshire Durham New Hampshire USA

**Keywords:** algae, coastal upwelling, competition, disturbance, grazing, light, rocky intertidal, top‐down/bottom‐up

## Abstract

Top‐down and bottom‐up factors and their interaction highlight the interdependence of resources and consumer impacts on food webs and ecosystems. Variation in the strength of upwelling‐mediated ecological controls (i.e., light availability and herbivory) between early and late succession stages is less well understood from the standpoint of influencing algal functional group composition. We experimentally tested the effect of light, grazing, and disturbance on rocky intertidal turf‐forming algal communities. Studies were conducted on the South Island of New Zealand at Raramai on the east coast (a persistent downwelling region) and Twelve Mile Beach on the west coast (an intermittent upwelling region). Herbivory, light availability, and algal cover were manipulated and percent cover of major macroalgal functional groups and sessile invertebrates were measured monthly from October 2017 to March 2018. By distinguishing between algal functional groups and including different starting conditions in our design, we found that the mosaic‐like pattern of bare rock intermingled with diverse turf‐forming algae at Twelve Mile Beach was driven by a complex array of species interactions, including grazing, predation, preemptive competition and interference competition, colonization rates, and these interactions were modulated by light availability and other environmental conditions. Raramai results contrasted with those at Twelve Mile Beach in showing stronger effects of grazing and relatively weak effects of other interactions, low colonization rates of invertebrates, and light effects limited to crustose algae. Our study highlights the potential importance of an upwelling‐mediated 3‐way interaction among herbivory, light availability, and preemption in structuring contrasting low rocky intertidal macroalgal communities.

## Graphical abstract

Variation in the strength of upwelling‐mediated ecological controls (i.e., light availability and herbivory) between early and late succession stages is less well understood from the standpoint of influencing algal functional group composition. Studies were conducted on the South Island of New Zealand at Raramai on the east coast (a persistent downwelling region) and Twelve Mile Beach on the west coast (an intermittent upwelling region). Herbivory, light availability, and algal cover were measured. By distinguishing between algal functional groups and including different starting conditions in our design, we found that Twelve Mile Beach was driven by a complex array of species interactions, including grazing, predation, preemptive competition and interference competition, colonization rates, and these interactions were modulated by light availability and other environmental conditions. Raramai results contrasted with those at Twelve Mile Beach in showing stronger effects of grazing and relatively weak effects of other interactions, low colonization rates of invertebrates, and light effects limited to crustose algae.

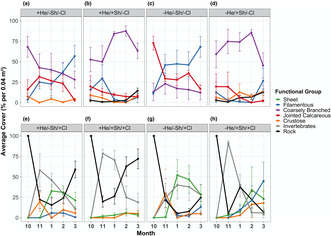

## 1 INTRODUCTION

**FIGURE 1:**
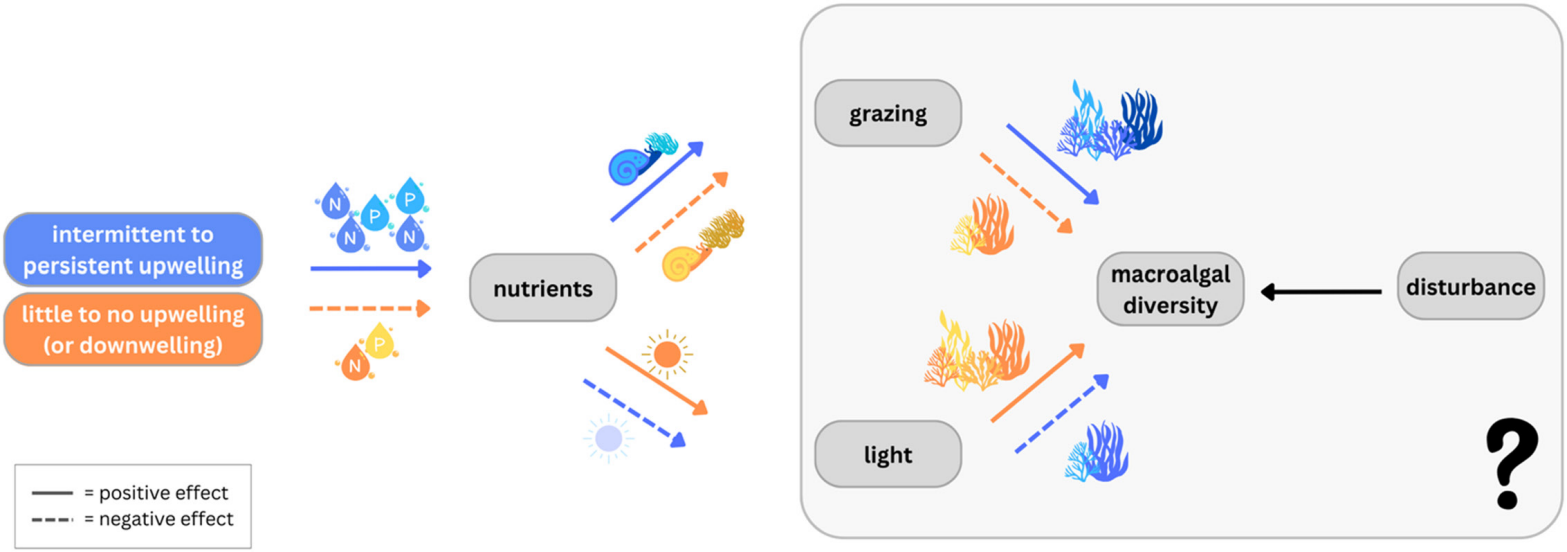
A summary of upwelling‐mediated effects on intertidal macroalgal diversity. Intermittent to persistent upwelling [colored blue]. Intermittent to persistent upwelling brings a large amount of nutrients to the surface (depicted by a solid line, indicating a positive effect). *Top path*: Increased nutrients will promote higher grazing activity on macroalgae, which in turn will increase the algal diversity. *Bottom path*: Increased nutrients will promote phytoplankton blooms and reduce the amount of light reaching the benthos, which in turn will reduce macroalgal diversity (depicted by a dashed line, indicating a negative effect). Little to no upwelling (or downwelling) [colored orange]. Little to no upwelling brings a limited amount of nutrients to the surface. *Top path*: Limited nutrients will decrease grazing activity on macroalgae, which in turn will reduce the algal diversity. *Bottom path*: Limited nutrients will allow a high amount of light to reach the benthos, which in turn will increase macroalgal diversity. Disturbance also plays a role in influencing macroalgal diversity by initiating algal succession. *The shaded region represents the unknown effects of a three‐way interaction among grazing, light availability, and disturbance (or preemption) on macroalgal communities, and the 3‐way interaction is the central question in our study*.

Top‐down and bottom‐up factors act in concert to influence diversity and composition of a community (Gruner et al., 2008; Hillebrand et al., 2007). The interaction between these two factors highlights the interdependence of resources and consumer impacts on food webs and ecosystems. In coastal environments, upwelling regimes can be important in modulating these interactions by underlying variation in temperature, nutrients, and light, which in turn can influence the strength and direction of species interactions (Bustamante, Branch, Eekhout, Robertson, et al., 1995; Hacker et al., 2019; Menge, 1992, 2000; Menge et al., 2015; Figure 1).

In eastern boundary coastal ecosystems, intermittent to persistent upwelling brings nutrient‐rich waters from the deep, which in turn promotes benthic macroalgal productivity (Bosman et al., 1987; Bustamante, Branch, Eekhout, Robertson, et al., 1995; Menge, 1992; Nielsen & Navarrete, 2004; Tapia et al., 2009; Wieters et al., 2009). High algal productivity is often associated with higher grazing activity (Menge et al., 1999) or greater average biomass of grazers (Bustamante, Branch, & Eekhout, 1995; Bustamante, Branch, Eekhout, Robertson, et al., 1995). Grazing activity has been documented to positively affect producer community structure in highly productive (e.g., high nutrient) environments (Hillebrand et al., 2007; Menge et al., 2023; Sellers et al., 2020). In such environments where rare species can now use new resources, herbivores can attenuate competitive effects by reducing competitively dominant species and promoting producer coexistence. However, grazing mostly negatively affects producers in unproductive (e.g., nutrient poor) environments (Hillebrand et al., 2007, Menge et al., 2023, Sellers et al., 2020). In low productivity environments, herbivores can negatively impact diversity by unselective removal of species or targeted removal of rare species.

In contrast, elevated nutrients from intermittent to persistent upwelling may indirectly increase the turbidity of the water column by stimulating phytoplankton blooms and subsequently shading the benthos (Kavanaugh et al., 2009) and limiting macroalgal growth and productivity (Kavanaugh et al., 2009; Spiecker & Menge, 2022). Alternatively, shading (e.g., phytoplanktom blooms, canopy cover) may facilitate macroalgal diversity by increasing water retention and limiting competitive exclusion among species (Eriksson et al., 2006; Watt & Scrosati, 2013).

Furthermore, disturbance‐driven ecological succession may affect producer community diversity (Noël et al., 2009; Pfeiffer et al., 2015; Sousa, 1979). During early successional stages, environmental factors (e.g., space and light) interact with disturbance and play an important role in establishing the community. With plentiful light and newly opened spaces, early successional species such as foliose algae are able to rapidly colonize the disturbed spaces. As soon as species are established, biological controls (e.g., grazing and competition) enter the picture. Grazers may interact with disturbance to alter the rate of succession; depending on their preference, they may either remove early successional species (Lubchenco, 1986; Lubchenco & Gaines, 1981) or later successional species (Sousa et al., 1981).

In addition to grazing, light availability, and disturbance, algal morphological and functional differences may shape algal community composition. For example, by impeding grazer activity and retaining water, the turf forms of certain intertidal algae lessen mortality from herbivory and desiccation (Hay, 1981). Specialized internal and external structures of opportunistic fleshy algae help them occupy ephemeral or newly disturbed habitats (e.g., Vermeij, 1978) or confer adaptive advantages under various light regimes (e.g., Ramus, 1978). Crustose grazer‐resistant stages of life histories of frondose algae, such as *Gigartina* and *Scytosiphon* (Littler & Littler, 1980, 1983; Lubchenco & Cubit, 1980; Slocum, 1980) increase survival under conditions of high herbivory and possibly intense physical disturbances (e.g., sand‐scour; Littler & Littler, 1983).

The effects of 1‐way and 2‐way interactions between grazing, light availability, and disturbance on macroalgal community composition are well studied. However, the effects of upwelling‐mediated three‐way interactions (grazing × light × disturbance) filtered by morphological and functional differences among macroalgal species remains insufficiently explored from the standpoint of influencing algal community composition (Menge et al., 2021).

Because of advantageous spatial and temporal scales, rocky shores are useful model systems for addressing the impacts of these abiotic and biotic interactions (Connell, 1972; Paine & Fenchel, 1994; Paine & Gould, 1977). Rocky shores in New Zealand are occupied by an exceptional diversity and abundance of macroalgae (Nelson, 2020; Schiel, 1990), and on the South Island, are characterized by contrasting oceanic conditions (Menge et al., 1999, 2003; Stevens et al., 2021). The west coast experiences intermittent upwelling with higher nutrient inputs and phytoplankton levels, and the downwelling‐dominated east coast experiences little upwelling with lower nutrient inputs and phytoplankton levels (Menge et al., 1999, 2003; Menge & Menge, 2013; Stevens et al., 2021). With these natural geophysical differences between both coasts of the South Island in mind, we asked the following questions to delineate the individual and interactive effects of grazing, light availability, and disturbance on macroalgal functional groups:

1. What is the relationship between grazing and responses of each macroalgal (juveniles and adults combined) functional group? We hypothesized that molluscan herbivores (limpets, chitons; the dominant herbivores in this system—Dunmore & Schiel, 2003; Guerry et al., 2009; Guerry & Menge, 2017) preferentially graze fleshy macroalgae, thereby allowing more resistant groups to increase in abundance (e.g., Lubchenco, 1978).
2. What is the relationship between light availability and responses of each macroalgal functional group? We hypothesized that reduced light (shading) will lower the abundance of all functional groups.
3. Do light availability and grazing differ in their effects on established algae (uncleared plots) compared to algae colonizing disturbed plots (cleared plots)? We hypothesized that in cleared plots, the effects of grazing and light availability will be stronger because (i) herbivores preferentially graze on fleshy algae, many of which are early colonizers (Lubchenco, 1978), (ii) more space is available during the early successional stages, meaning the area will be largely exposed to light, and (iii) molluscan grazers are often deterred by rugose substrata such as that found in turf‐forming algae (Creese, 1982).

## 2 MATERIALS AND METHODS

### 2.1 Study system

**FIGURE 2:**
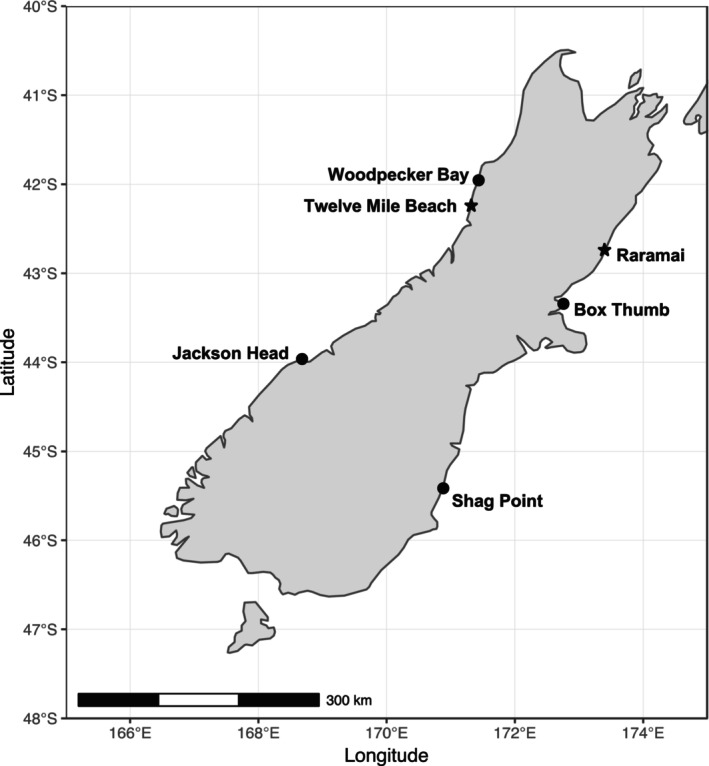
Map of the two study sites, Raramai (−42.46° S, 173.55° E) and Twelve Mile Beach (−42.82° S, 171.82° E) on opposite coasts of the South Island of New Zealand. Stars = our study sites and circles = other long‐term monitoring sites (Menge et al., 1999, 2003).

**FIGURE 3:**
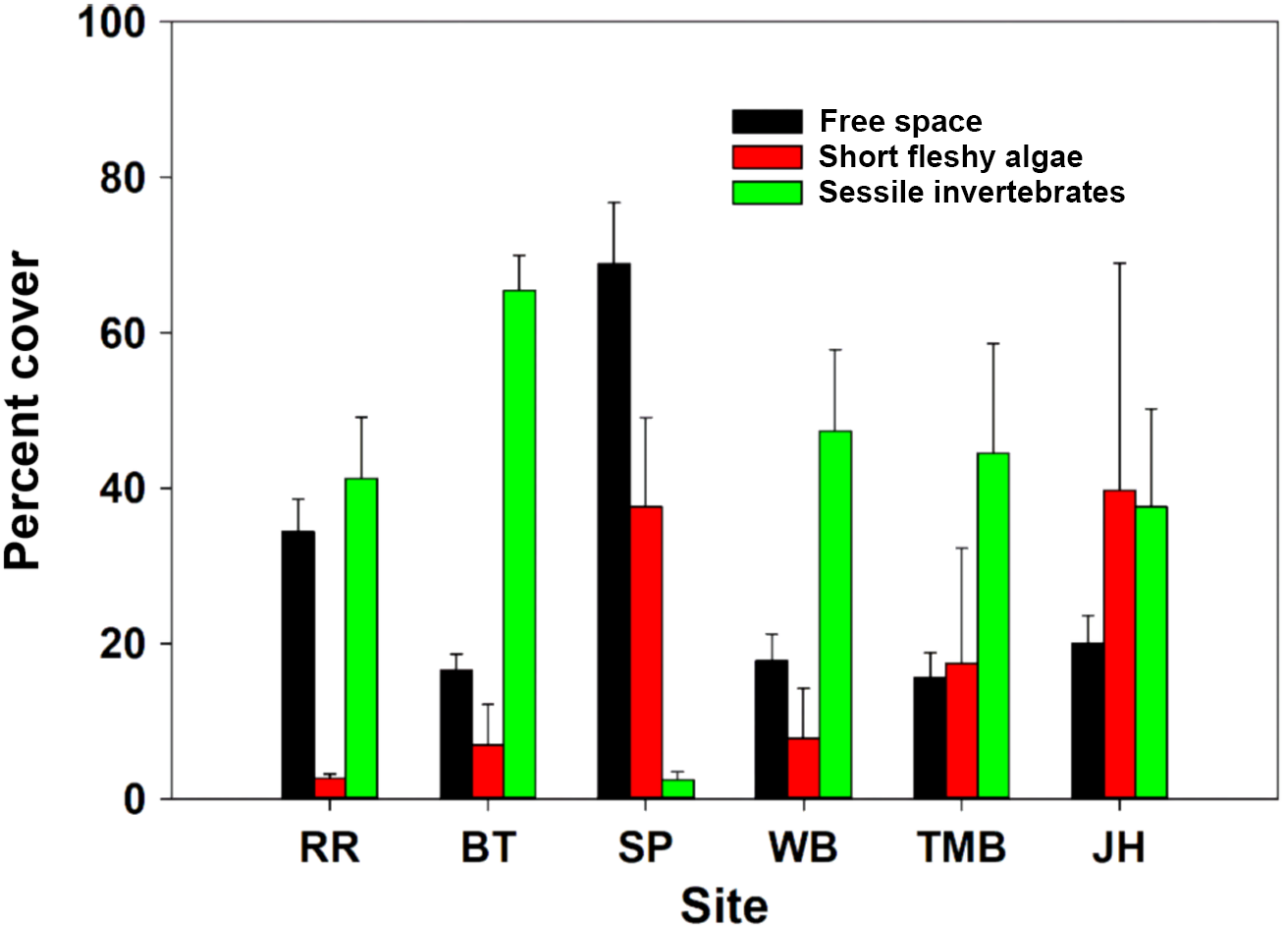
Average abundance of free space (bare rock plus crustose algal cover), “short” fleshy algae (turf‐forming algae, filamentous algae, sheet‐forming algae), and sessile invertebrates (mostly mussels and barnacles) at six New Zealand study sites. Site codes are RR = Raramai, BT = Box Thumb, SP = Shag Point, WB = Woodpecker Bay, TMB = Twelve‐Mile Beach, and JH = Jackson Head. Data are mean ± 1 standard error of the mean, and are overall averages across zones from periodic surveys taken between 1995 and 2010.

This research was conducted in the context of a well‐studied rocky intertidal meta‐ecosystem on the South Island of New Zealand (Menge et al., 1999, 2003, Schiel, 2004, 2011, Schiel et al., 2016, Menge & Menge, 2013, Rilov & Schiel, 2006a, 2006b, 2011; Figure 2). Wave‐exposed rocky shores on opposite coasts experience contrasting oceanic conditions, with intermittent upwelling regimes on the west coast and downwelling regimes on the east coast. We selected Raramai on the east coast and Twelve Mile Beach on the west coast to test the effects of upwelling‐mediated 3‐way interaction on intertidal macroalgal communities (Figures 2 and 3). Prior research has shown that nutrient inputs, sediment suspension, rates of recruitment, sessile invertebrate growth, phytoplankton abundance, and predation are higher at Twelve Mile Beach and lower at Raramai (Bracken et al., 2012; Menge et al., 1999, 2003; Menge & Menge, 2013). Despite the differences in upwelling regimes, these sites have similar levels of wave exposure (Menge et al., 1999).

### 2.2 Experimental design

At both sites 20 × 20 cm plots were established in the low zone, where turf‐forming macroalgae were abundant. The fully‐crossed randomized block experiment consisted of three treatments—light (shading), herbivory, and preemptive competition—with four replicates or blocks at each site (Figure A1). Each treatment had two levels: light reduction (shaded and unshaded), herbivory (present and absent), and pre‐emptive competition (cleared and uncleared).

Light level (shade) was manipulated using black plastic Vexar mesh (12‐mm mesh size, neutral spectral density). The mesh imitated natural shading by phytoplankton in the water column and attenuated light levels reaching benthic surfaces (e.g., Bertness et al., 1999; Kavanaugh et al., 2009). Mesh covers were fastened to the rocks using stainless‐steel lag screws inserted through plastic washers, the mesh and wall anchors placed in predrilled holes. Covers were domed, leaving space beneath them to minimize abrasion of swaying macroalgae when underwater. Due to logistical constraints, we were not able to employ mesh controls; however, sufficient previous studies show that employing herbivore exclusion mesh (with mesh size smaller than ours) did not introduce any artifacts (e.g., Bertness et al., 1999; Burnaford, 2004; Nielsen, 2001).

To quantify light levels in shaded vs. unshaded plots, at each site, we placed HOBO light/temperature sensors (ONSET Computer Corp., Part #: UA‐002‐64) beneath the mesh in each replicate of shaded treatments and near each replicate of unshaded treatments Light/temperature sensors recorded at 30‐min intervals. Using the resulting data, we estimated that during a full tidal cycle (including immersion and emersion periods), mesh attenuated light levels an average of 66% and 78% (i.e., 100 − [mean shaded light intensity/mean unshaded light intensity] × 100) throughout the experiment at Raramai and Twelve Mile Beach, respectively.

Herbivory was manipulated by coating a square band of Z‐spar marine epoxy (Koppers Splash Zone A‐788 compound) placed around each plot with copper‐based antifouling paint (Pettit Trinidad SR Antifouling Bottom Paint). Previous research has shown that such paint excludes “flat” grazers such as limpets and chitons (Cubit, 1984; Farrell, 1988; Menge, 2000; Menge et al., 1999; Paine, 1984; Sousa, 1979). Preemption was tested by clearing half of the plots of algae and invertebrates with a chisel and a wire brush. As is commonly done, the plots were sprayed with oven cleaner to remove algal crusts and diatoms still adhering to the rock (Cubit, 1984; Freidenburg et al., 2007; Menge, 2000). When waves cover oven cleaner in plots, the reaction converts the cleaner to NaCl and H_2_O, so the cleaner is unlikely to have lingering effects on the plots (Menge et al., 1999). The experiments were monitored monthly, and when damaged, shades were repaired and fouling organisms were removed.

To maximize time available for experimental setup and monitoring at the low intertidal level of these experiments, we did not establish paint controls. Prior experiments in this system that included such controls never found paint artifacts associated with these treatments (e.g., Guerry & Menge, 2017; Menge et al., 1999). Hence, we believe this decision did not affect the robustness of our results.

### 2.3 Biological measurements

Experiments ran during austral spring and summer, October 2017 to March 2018. Monthly photographs of each plot were used to quantify macroalgal cover using photoQuad software (Trygonis & Sini, 2012). Data were sorted into six macroalgal functional groups according to the Littler and Littler (1984) functional form scheme (Sheet, Filamentous, Coarsely Branched, Thick Leathery, Jointed Calcareous, and Crustose). We also attempted to categorize algae by their respective classes (Chlorophyta, Rhodophyta, and Phaeophyta), but due to the difficulty of distinguishing red and brown macroalgae via photos, we combined these two classes into one category (“Brown/Red”) in our analyses. In the photos, light‐ or desiccation‐damaged macroalgal functional groups (excluding crustose algae) that turned white were quantified as “Bleached Upright.” Similarly damaged crustose algae and bare rock were quantified as “Rock.” Space under limpets and chitons also was defined as “Rock” since when nonexperimental animals were removed, the substratum underneath them usually was bare. In addition to quantifying macroalgal functional groups, we also quantified space‐occupying sessile invertebrates (i.e., barnacles and mussels).

### 2.4 Statistical analyses

To assess the effects of herbivory, light availability, and preemptive competition on macroalgal community composition, we used permutational analysis of variance (PERMANOVA), permutational dispersion (PERMDISP), and hierarchical linear mixed model (HLMM). PERMANOVA was used to analyze the effects of herbivory, preemption, and light availability on the overall community composition through time. Data were square‐root transformed for analysis, and these values were then converted to Bray–Curtis similarity for generation of resemblance matrices. The analysis used partial Type III sums of squares, and fixed effects summed to zero for mixed terms. The permutation method used residuals under a reduced model, and we ran 9999 permutations. Estimates of components of variation (in squared units of Bray–Curtis dissimilarity) are provided by PERMANOVA; we took the square root of these to put them back into Bray–Curtis units and used these as measures of explained variability.

We conducted two levels of analysis, a repeated measures PERMANOVA on the entire dataset (i.e., functional group responses across all treatment combinations, times and sites), and tests of herbivory and light separately for each site × preemption treatment. In the full model, site, herbivory, light, preemption, month of experiment, and all interactions were fixed effects, block was a random effect, and cover of functional groups was the response variable. In the site × preemption analyses, final cover of functional groups was the response variable. Finally, we used PERMDISP to test if the dispersions between groups varied significantly.

HLMM was used to analyze the effects of herbivory and light availability on individual macroalgal functional groups. We ran models separately for each preemption treatment. Restricted maximum likelihood and Kenward–Roger approximation corrections were used to minimize small sample size bias and prevent inflation of Type‐I error rates. Site, treatment, and the site × treatment interactions were fixed effects, and block was a random effect. The response variable, final cover, was log_10_(*x* + 1)‐transformed. These transformed values were back‐transformed to acquire interpretable least squares means and standard error. The delta method was used for the back transformation of standard error (Ver Hoef, 2012). Note that any functional group with abundance <5% in cover throughout the experiment was excluded from analyses. Model assumptions appropriate for each analysis (independence, homoscedasticity, and normality) were examined visually and in all cases, the data met the criteria.

Least squares means (LSM) from the HLMM model were used for multiple pairwise comparisons. Corrections were not applied for these comparisons because the contrasts were planned a priori with an intention of comparing the observed results with prior results in the literature. Furthermore, reducing the type I error for null associations may increase the type II error for those associations that are not null, which is a concern when important differences may be deemed nonsignificant (Feise, 2002; Perneger, 1998; Rothman, 1990). Instead of applying corrections, we report precise *p*‐value and standard error. Below, we report both untransformed mean percent cover ±1 SE and transformed (log_10_[LSM Estimate] mean ± 1 SE). To reduce clutter, in the results we refer to the latter as “transformed means.” For clarity in interpretation, we plotted means of raw percent cover in the figures, but did our statistical analyses on LSM values, which also allowed adjusting for means of other factors in the model.

Software and code used for PERMANOVA analyses were PRIMER PERMANOVA+ (PRIMER‐e 2017, Version 7) and for HLMM analyses were SAS Enterprise Guide (SAS Institute Inc. 2013, Version 7.1, Procedure: MIXED).

Lastly, we used linear regression to analyze potential competition between macroalgal functional groups at TMB. We deleted all cases in which abundance of both functional groups was zero and used only data from uncleared plots since much space in cleared plots was bare during the experiments. Based on the critique by Warton and Hui (2011), we ran separate analyses using arcsin and logit transformations to test which gave better results. Software and code used for the analysis were R Studio (R Studio 2018, Version 1.1.456, Package: Base; Functions: lm and cor).

## 3 RESULTS

Our experiment showed evidence of significant individual and interactive effects of herbivory, light availability and preemption on algal functional group composition at each site (PERMANOVA; Table 1, herbivory × site [*p* = .0001], light × site [*p* = .0001], preemption × site [*p* = .0001], herbivory × light × preemption × site [*p* = .0001]). Below, we disentangle the effects of each factor.

**TABLE 1:** PERMANOVA: repeated measures test of effects of site, herbivory, light, preemption by month, and block on macroalgal community composition during the experiment.

Effect	DF	SS	MS	Pseudo‐*F*	*p*‐value	# of unique permutations	%variance explained
Site	1	100,870	100,870	138.84	**.0001**	9960	11.21
Block	3	8876.6	2958.9	4.07	**.0001**	9935	0.48
Herbivory	1	29,129	29,129	40.09	**.0001**	9960	3.18
Light	1	7556	7556	10.4	**.0001**	9961	0.76
Preemption	1	173,760	173,760	239.17	**.0001**	9959	19.36
Month	4	28,848	7212.1	9.93	**.0001**	9944	1.80
Herbivory × Site	1	9094.3	9094.3	12.52	**.0001**	9963	1.87
Light × Site	1	13,010	13,010	17.91	**.0001**	9964	2.75
Preemption × Site	1	75,675	75,675	104.16	**.0001**	9951	16.78
Month × Site	4	18,708	4677.1	6.44	**.0001**	9936	2.20
Herbivory × Light	1	2331.4	2331.4	3.21	**.024**	9961	0.36
Herbivory × Preemption	1	3827.7	3827.7	5.27	**.002**	9968	0.69
Herbivory × Month	4	10,115	2528.7	3.48	**.0002**	9966	1.00
Light × Preemption	1	2043.3	2043.3	2.81	**.046**	9966	0.29
Light × Month	4	7688.1	1922	2.65	**.0019**	9927	0.66
Preemption × Month	4	46,163	11,541	15.89	**.0001**	9937	6.03
Herbivory × Site × Light	1	2080.4	2080.4	2.86	**.04**	9963	0.61
Herbivory × Site × Preemption	1	652.39	652.39	0.90	.48	9950	−0.03
Herbivory × Site × Month	4	6077.2	1519.3	2.09	**.019**	9926	0.88
Light × Site × Preemption	1	8865.5	8865.5	12.4	**.0001**	9958	3.64
Light × Site × Month	4	4341.4	1085.4	1.49	.13	9927	0.40
Preemption × Site × Month	4	12,891	3222.6	4.44	**.0001**	9938	2.78
Herbivory × Light × Preemption	1	1512.9	1515.9	2.08	.11	9964	0.35
Herbivory × Light × Month	4	4419.3	1104.8	1.52	.13	9921	0.42
Herbivory × Preemption × Month	4	2144.8	536.19	0.74	.70	9944	−0.21
Light × Preemption × Month	4	5599.7	1399.9	1.93	**.035**	9923	0.75
Herbivory × Light × Preemption × Site	1	9075.5	9075.9	12.49	**.0001**	9964	7.46
Herbivory × Light × Site × Month	4	2788.3	697.08	0.96	.50	9947	−0.07
Herbivory × Preemption × Site × Month	4	1276.2	319.06	0.44	.92	9911	−0.90
Light × Preemption × Site × Month	4	5551.4	1387.8	1.91	**.034**	9922	1.47
Residual	265	192,530	726.52				13.02
Total	339	820,550					

*Note*: Bold values are statistically significant *p*‐values (*p* < 0.05).

### 3.1 Overall algal abundance in experiments

**FIGURE 4:**
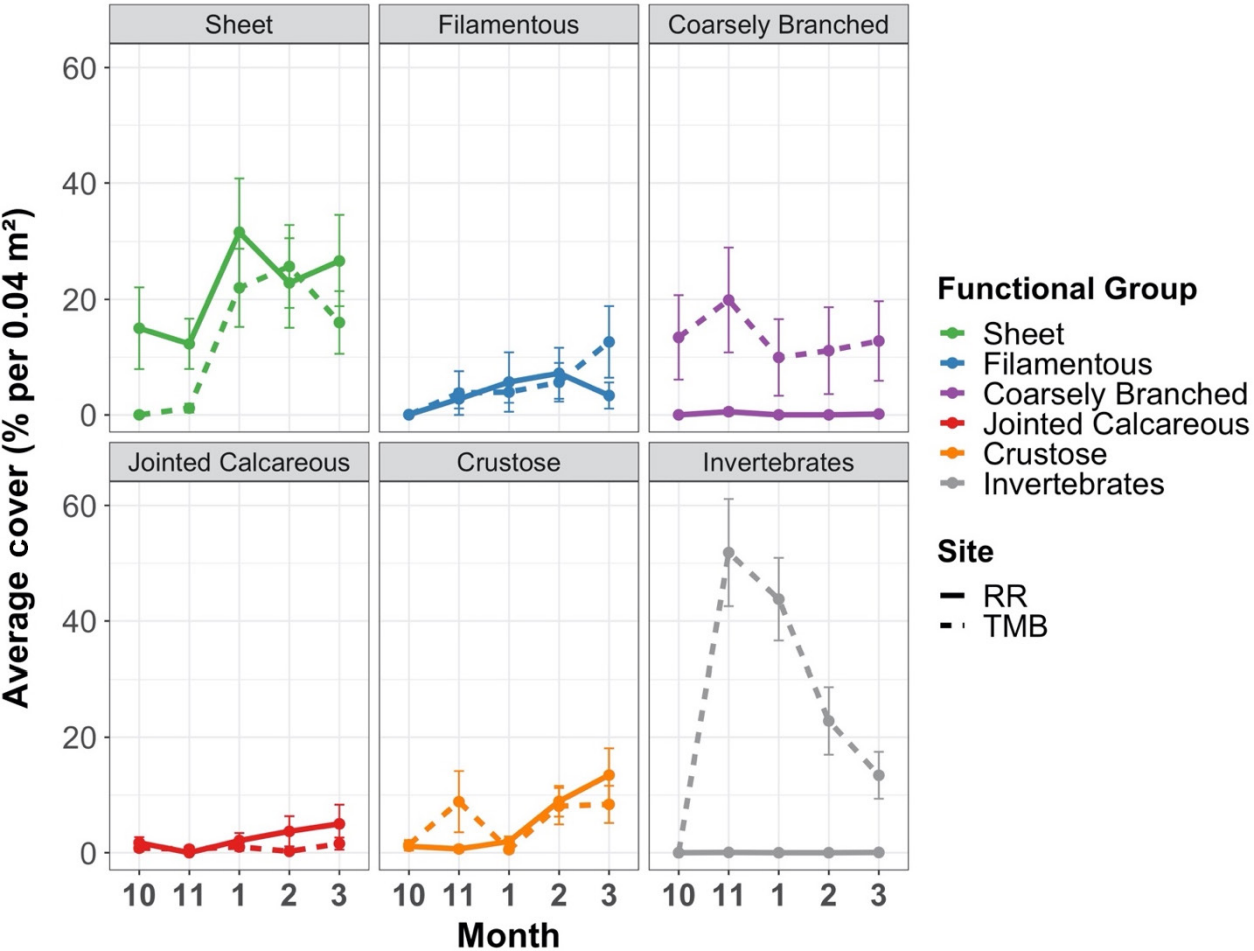
Average percent cover (all treatments combined) of macroalgal functional groups across months in the cleared plots in Raramai and Twelve Mile Beach. All values are arithmetic means ± standard error. Each panel corresponds to a macroalgal functional group. The site abbreviations and line type are: RR = Raramai (solid line) and TMB = Twelve Mile Beach (dashed line). Macroalgal functional group line codes are: Sheet = green, Filamentous = blue, Coarsely Branched = purple, Jointed Calcareous = red, Crustose = orange, Invertebrates = gray.

**FIGURE 5:**
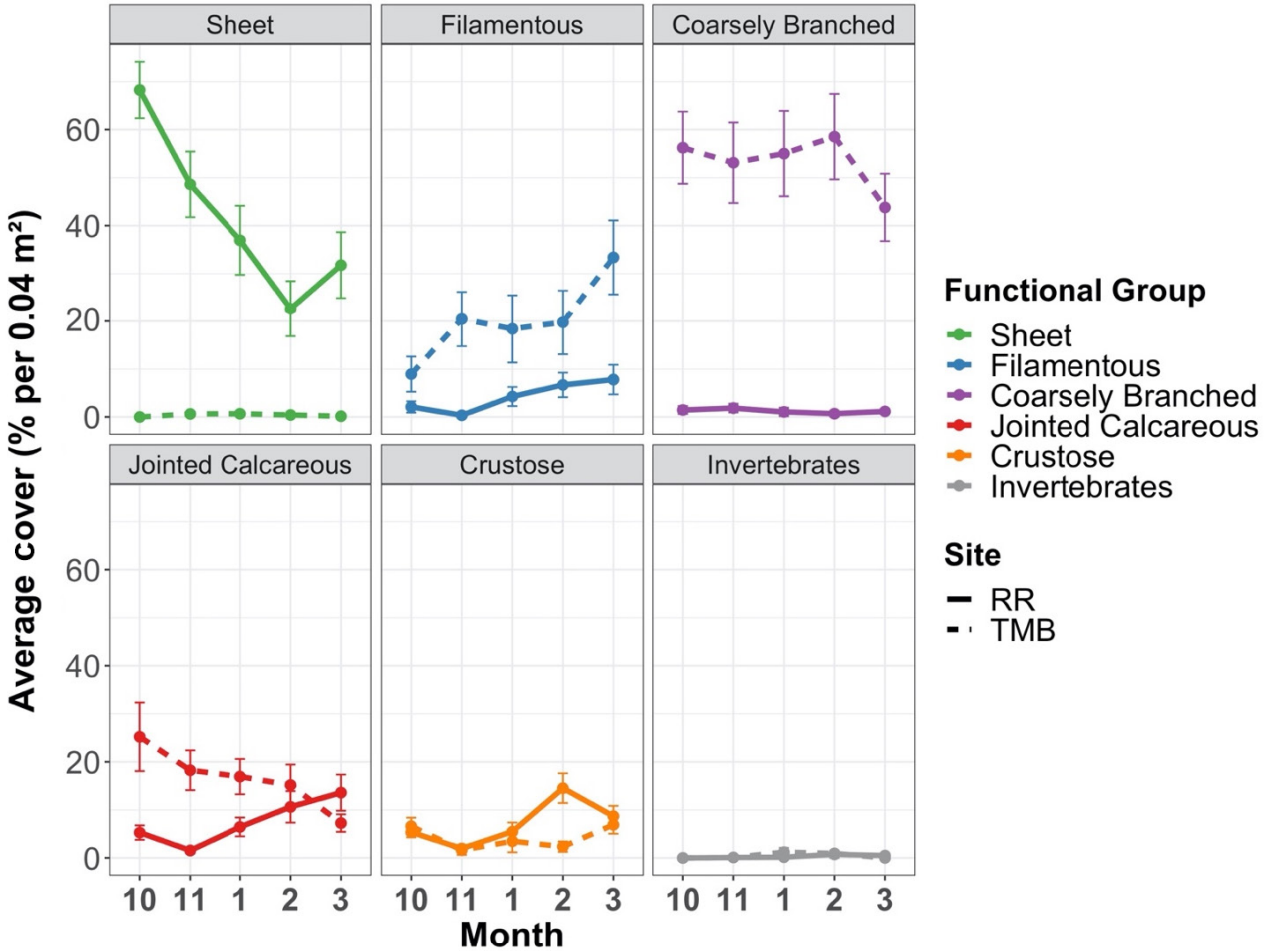
Average percent cover (all treatments combined) of macroalgal functional groups across months in the uncleared plots at Raramai and Twelve Mile Beach. All values are arithmetic mean ± standard error. Each panel corresponds to a macroalgal functional group. Site and color codes are as in Figure 4.

In the overall analysis, multivariate dispersion for algal functional group composition was heterogeneous between sites and preemption treatments but was homogeneous among blocks, herbivory and light (PERMDISP; Table 2). In cleared plots, covers of sheet, filamentous, jointed calcareous, and crustose macroalgae were similar between sites, but Twelve Mile Beach had higher covers of coarsely branched macroalgae and invertebrates (Figure 4). In uncleared plots, both sites had comparable covers of crustose macroalgae and invertebrates, but Raramai had more sheet macroalgae while Twelve Mile Beach had more filamentous, coarsely branched, and jointed calcareous macroalgae (Figure 5). By phylum, Raramai had mostly green sheet and filamentous algae while sheet and filamentous algae at Twelve Mile Beach were mostly browns and reds (Figure A2).

**TABLE 2:** PERMDISP results testing multivariate dispersion in the PERMANOVA analyses.

Analysis	Factor	Number of groups	Number of samples	df	*F*	*p*(perm)
All data	Site	2	340	1338	85.6	**.001**
	Block	4	340	3336	1.69	.28
	Herbivory	2	340	1338	3.55	.11
	Light	2	340	1338	2.49	.18
	Preemption	2	340	1338	22.3	**.001**
Raramai cleared	Herbivory	2	16	1,14	4.63	.062
	Light	2	16	1,14	0.61	.46
Raramai uncleared	Herbivory	2	20	1,18	0.31	.59
	Light	2	20	1,18	0.26	.63
Twelve mile beach cleared	Herbivory	2	16	1,14	8.81	**.005**
	Light	2	16	1,14	11.49	**.01**
Twelve mile beach uncleared	Herbivory	2	19	1,17	0.87	.38
	Light	2	19	1,17	2.22	.15

*Note*: Analyses used Bray‐Curtis similarities in resemblance matrices after square root transformations of functional group abundances. Number of permutations was 999 for the “all data” test and 9999 for the site × disturbance tests, and the tests analyzed deviations from the centroid for each factor.

Bold values are statistically significant *p*‐values (*p* < 0.05).

### 3.2 Effects of herbivory and light availability in cleared and uncleared plots by site: Raramai

**FIGURE 6:**
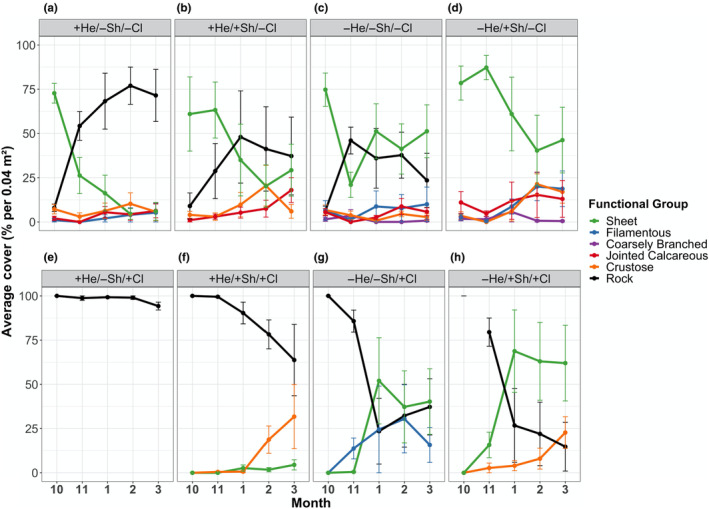
Average percent cover of macroalgal functional groups at Raramai throughout the experiment for eight different treatments. All values are reported using arithmetic mean ± standard error. Each panel corresponds to a treatment. The top row shows uncleared plot (−Cl) results, and the bottom row shows cleared plot (+Cl) results. Herbivore effects are shown by comparing the first two columns (+He) to the last two columns (−He). Shading effects are shown by comparing the first column (−Sh) to the second (+Sh), and the third (−Sh) to the fourth (+Sh). Macroalgal functional group line codes are: Sheet = green, Filamentous = blue, Coarsely Branched = purple, Jointed Calcareous = red, Crustose = orange, Rock = black. Functional groups with cover <5% for all months are not shown.

At Raramai, herbivores altered macroalgal community composition in cleared plots (variance components = 33.9; Table 3, Pseudo *F* = 13.1, *p* = .004), but had weaker effects in uncleared plots (variance components = 16.9; Table 4, Pseudo *F* = 2.96, *p* = .058). For example, in cleared plots, herbivores strongly reduced the cover of sheet algae. In unshaded *+herbivore* plots, final percent cover of sheets was 39.75 ± 18.09% lower than in unshaded −*herbivore* plots (transformed estimate = 21.35 ± 7.12%; [+He/−Sh/+Cl vs. −He/−Sh/+Cl] *p* = .0001; Figure 6e,g; Table A1). Similarly, in shaded *+herbivore* plots, final percent cover of sheets was 57.50 ± 20.50% lower than in shaded *−herbivore* plots (transformed estimate = 8.90 ± 2.96%; [+He/+Sh/+Cl vs. −He/+Sh/+Cl] *p* = .005; Figure 6f,h; Table A1).

**TABLE 3:** PERMANOVA: raramai cleared plots.

Effect	DF	SS	MS	Pseudo‐*F*	*p*‐value	# of unique permutations	Sq. root of var. components estimate
Block	3	3942	1314	1.73	.19	9953	11.76
Herbivory	1	9935.7	9935.7	13.06	**.004**	9954	33.87
Light	1	5146.1	5146.1	6.76	**.014**	9963	23.41
Herbivory × Light	1	580.66	580.66	0.76	.51	9938	−6.72
Residual	9	6850.5	761.17				27.59
Total	15	26,455					

*Note*: Testing macroalgal community composition at the end of the experiment.

Bold values are statistically significant *p*‐values (*p* < 0.05).

**TABLE 4:** PERMANOVA: raramai uncleared plots.

Effect	DF	SS	MS	Pseudo‐*F*	*p*‐value	# of unique permutations	Sq. root of var. components estimate
Block	3	3176.2	1058.7	0.80	.62	9939	−7.36
Herbivory	1	3934.4	3934.4	2.96	.058	9954	16.88
Light	1	1288.5	1288.5	0.97	.41	9954	−2.11
Herbivory × Light	1	954.98	954.98	0.72	.56	9962	−9.05
Residual	13	17,281	1329.3				36.46
Total	19	27,336					

*Note*: Testing macroalgal community composition at the end of the experiment.

In uncleared plots, the effect of herbivory on sheets was weaker, affecting only the unshaded plots. Specifically, in unshaded *+herbivore* plots, the final percent cover of sheet macroalgae was 45.25 ± 15.52% lower than unshaded *−herbivore* plots (transformed estimate = 14.00 ± 4.55%; [+He/−Sh/−Cl vs. −He/−Sh/−Cl] *p* = .06; [+He/+Sh/−Cl vs. −He/+Sh/−Cl] *p* = .91; Figure 6a–d; Table A2).

At Raramai, light availability affected community composition in cleared plots (variance components = 23.4) but not in uncleared plots (variance components = −2.11; Tables 3 and 4; Pseudo *F* = 6.76 and 0.97, and *p* = .014 and .41, respectively). The test of homogeneity was met for both cleared and uncleared plots at Raramai (PERMDISP; Table 2). Light availability had a particularly strong negative effect on crustose algae. For example, in *shaded* + herbivore plots, final cover of crustose algae was 31.50 ± 18.26% higher than in *unshaded* + herbivore plots (transformed estimate = 10.77 ± 3.35%; [+He/−Sh/+Cl vs. +He/+Sh/+Cl] *p* = .0023; Figure 6e,f; Table A1). Similarly, in *shaded* −herbivore plots, final crustose algae cover was 22.25 ± 39.34% higher than in *unshaded* −herbivore plots (transformed estimate = 11.38 ± 3.79%; [−He/−Sh/+Cl vs. −He/+Sh/+Cl] *p* = .0018; Figure 6g,h; Table A1).

In uncleared plots, light (unshaded plots) had no effect on individual functional groups except for crustose algae. The effect of light on crustose algae was weaker in uncleared plots than in cleared plots, affecting only −herbivore plots. In *shaded* −herbivore plots, final crustose algal cover was 17.00 ± 6.42% higher than in *unshaded* −herbivore plots (transformed estimate = 5.14 ± 1.67%; [−He/−Sh/−Cl vs. −He/+Sh/−Cl] *p* = .0304; Figure 6c,d; Table A2). Furthermore, though not significant, jointed calcareous algal cover in Raramai *shaded* plots tended to increase more than in *unshaded* plots (Figure 6a–d).

### 3.3 Effects of herbivory and light availability in cleared and uncleared plots by site: Twelve Mile Beach

**FIGURE 7:**
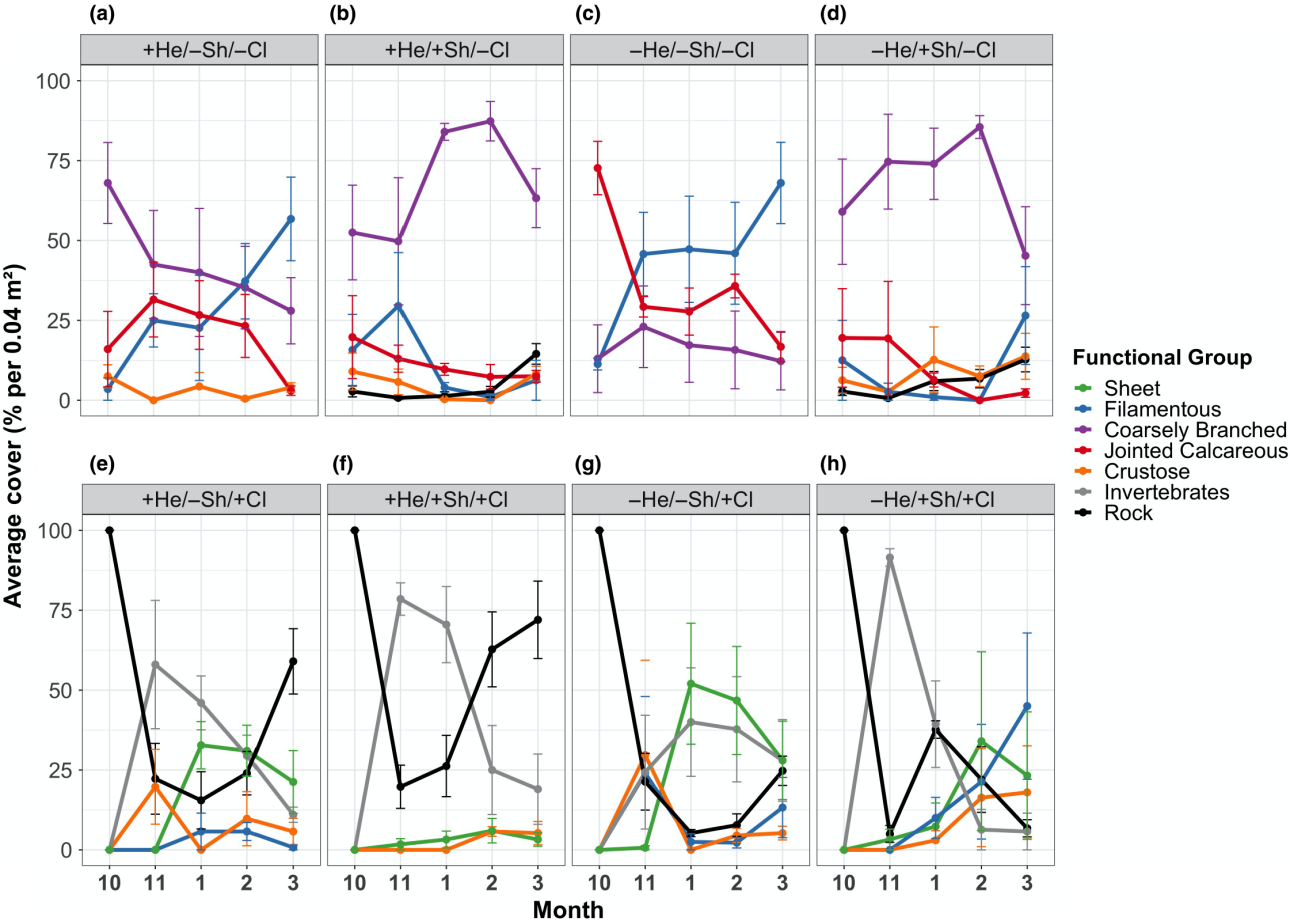
Average percent cover of macroalgal functional groups in Twelve Mile Beach throughout the experiment for eight different treatments. All values are reported using arithmetic mean ± standard error. Each panel corresponds to a treatment. Layout with respect to treatments is the same as that in Figure 6. Macroalgal functional group line codes are the same as in Figure 6 caption with the addition of Invertebrates = gray.

**FIGURE 8:**
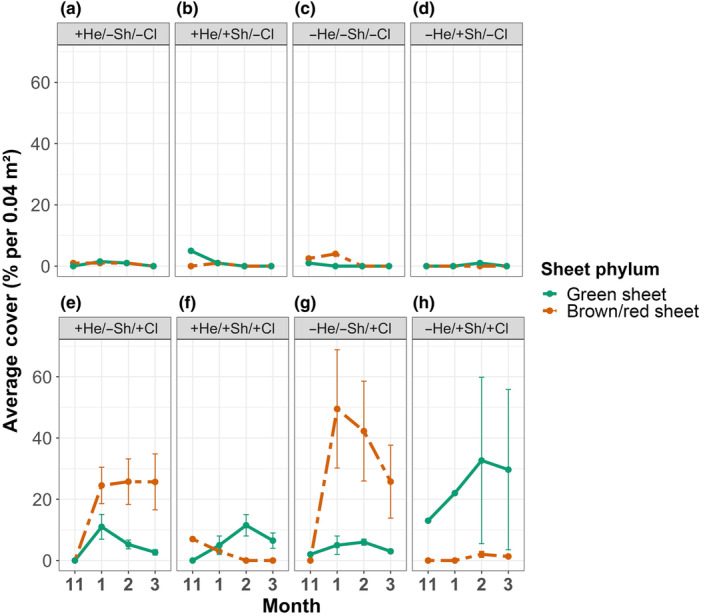
Average percent cover of sheet algae at Twelve Mile Beach throughout the experiment for eight different treatments. All values are reported using arithmetic mean ± standard error. Each panel corresponds to a treatment. The treatment layout is the same as in Figure 6. Sheet phylum group line codes are: Green Sheet = green solid, Brown/Red Sheet = orange dot‐dash.

At Twelve Mile Beach, herbivory was the only factor shaping community composition in cleared plots (variance components = 26.2) (Table 5, Pseudo *F* = 6.8, *p* = .004). In this case, the assumption of homogeneity of multivariate dispersion was not met (PERMDISP; Table 2). In shaded −*herbivore* plots, final filamentous algal cover was 45.00 ± 22.88% higher than in shaded *+herbivore* plots (transformed estimate = 17.47 ± 5.81%; [+He/+Sh/+Cl vs. −He/+Sh/+Cl] *p* = .0003; Figure 7f,h; Table A1). In contrast to the strong effect on sheet algae seen at Raramai, herbivores had no effect on sheet algae at Twelve Mile Beach (*p* = .14, Table A1). At this site, sheets were mostly absent in uncleared plots, but were abundant in cleared plots (Figure 7a–h). Separation of sheets by phyla reveals that brown/red sheets were only abundant in unshaded treatments, while green sheets were present in all treatments (Figure 8e–h). The cover of green sheets was highest in shaded −herbivore plots. Cover of green sheets in unshaded +herbivore plots gradually decreased over time while cover of brown/red sheets remained constant (Figure 8e).

**TABLE 5:** PERMANOVA: twelve mile beach cleared plots.

Effect	DF	SS	MS	Pseudo‐*F*	*p*‐value	# of unique permutations	Sq. root of var. components estimate
Block	3	3515.2	1171.7	1.24	.32	9937	7.55
Herbivory	1	6417.5	6417.5	6.80	**.004**	9944	26.16
Light	1	1258.8	1258.8	1.33	.30	9943	6.27
Herbivory × Light	1	1252.4	1252.4	1.33	.30	9958	8.78
Residual	9	8494.6	943.84				30.72
Total	15	20,938					

*Note*: Testing macroalgal community composition at the end of the experiment.

Bold values are statistically significant *p*‐values (*p* < 0.05).

Conversely, in uncleared plots, light availability and herbivory were the main factors in shaping community composition (variance components = 21.3 and 14.5, respectively; Table 6, pseudo *F* = 10.5 and 5.4, *p* = .003 and .038). Here the assumption of homogeneous multivariate dispersion was met (PERMDISP; Table 2). However, replicates (blocks) differed in this treatment × site (Pseudo *F* = 4.62, *p* = .009). At the functional group level, shading strongly decreased the cover of filamentous macroalgae. That is, in *shaded* + herbivore plots, final cover of filamentous macroalgae was 50.50 ± 14.75% lower than in *unshaded* + herbivore plots (transformed estimate = 23.35 ± 7.60%; [+He/−Sh/−Cl vs. +He/+Sh/−Cl final] *p* < .0001; Figure 7a,b; Table A2) and 41.50 ± 14.20% lower in *shaded* –herbivore plots than in *unshaded* –herbivore plots (transformed estimate = 8.72 ± 2.84%; [−He/−Sh/−Cl vs. −He/+Sh/−Cl] *p* = .0044; Figure 7c,d; Table A2). In contrast, shading increased coarsely branched algal cover, but only in −herbivore plots. In *shaded* −herbivore plots, coarsely branched algal cover was 33.00 ± 14.49% higher than in *unshaded* herbivore plots (transformed estimate = 6.25 ± 2.03%; [−He/+Sh/−Cl vs. −He/−Sh/−Cl] *p* = .0156; Figure 7c,d; Table A2).

**TABLE 6:** PERMANOVA model for macroalgal community composition in Twelve Mile Beach uncleared plots at the end of the experiment.

Effect	DF	SS	MS	Pseudo‐*F*	*p*‐value	# of unique permutations	Sq. root of var. components estimate
Block	3	5905.1	1968.4	4.62	**.009**	9956	18.09
Herbivory	1	2299.6	2299.6	5.40	**.038**	9974	14.49
Light	1	4490.4	4490.4	10.54	**.003**	9961	21.34
Herbivory × Light	1	1322.1	1322.1	3.10	.079	9963	14.47
Residual	12	5113.5	426.12				20.64
Total	18	18,334					

*Note*: Bold values are statistically significant *p*‐values (*p* < 0.05).

In addition to these herbivore and light effects, correlative evidence suggested that interspecific competition occurred between several macroalgal functional groups in uncleared plots at Twelve Mile Beach (Table 7; Figure 7). No correlations were significant at Raramai (data not shown). Specifically, filamentous algal abundance varied inversely with coarsely branched algal abundance (*r* = −.85, *p* < .0001), and jointed calcareous algae varied inversely with both coarsely branched (*r* = −.52, *p* < .0001) and crustose algae (*r* = −.62, *p* < .0001). Correlations between crustose vs. filamentous and coarsely branched algae were negative, but less than −.29. In contrast, filamentous and jointed calcareous algae were uncorrelated. [Note that arcsin and logit transformations gave similar results, with adjusted *R* ^2^ being higher for arcsin in three cases and higher for logit in three cases.] Furthermore, our observations indicated that sessile invertebrates competed with macroalgae for space. In early months, sessile invertebrates were abundant in cleared plots at Twelve Mile Beach and mostly preempted macroalgae in all treatments (Figure 7e–h). In later months, sessile invertebrate cover dropped in all treatments except for the unshaded—herbivore treatment. The cover was 22.25 ± 8.13% greater in *unshaded* −herbivore plots than in *shaded* −herbivore plots (transformed estimate = 6.49 ± 2.16%; [−He/+Sh/+Cl vs. −He/−Sh/+Cl] *p* = .0159; Figure 7g,h; Table A1).

**TABLE 7:** Linear regressions between macroalgal functional groups (all treatments combined) in Twelve Mile Beach uncleared plots.

Comparison	Transformation	Intercept	Slope	*F*	*p*	*N*	Adj. *R* ^2^	Corr. coef.
Filamentous vs. Coarsely Branched	ArcSin	1.11	−0.906	190.2	**<.0001**	73	.724	−0.851
	Logit	−2.42	−0.628	74.3	**<.0001**	73	.505	−0.710
Filamentous vs. Jointed Calcareous	ArcSin	0.42	0.051	0.09	.76	71	−.013	0
	Logit	−2.36	0.104	0.35	.55	71	−.01	0
Filamentous vs. Crustose	ArcSin	0.520	−0.664	9.02	**.003**	94	.079	−0.281
	Logit	−5.22	−0.550	19.67	**<.0001**	94	.167	−0.409
Coarsely Branched vs. Crustose	ArcSin	0.314	−0.162	9.25	**.003**	92	.083	−0.288
	Logit	−4.497	−0.218	6.62	**.012**	92	.059	−0.243
Coarsely Branched vs. Jointed Calcareous	ArcSin	1.103	−0.710	30.96	**<.0001**	81	.272	−0.522
	Logit	−0.836	−0.389	23.64	**<.0001**	80	.223	−0.472
Crustose vs. Jointed Calcareous	ArcSin	0.276	−0.371	51.71	**<.0001**	83	.382	−0.618
	Logit	−6.481	−0.641	68.70	**<.0001**	83	.452	−0.672

*Note*: Bold values are statistically significant *p*‐values (*p* < 0.05).

## 4 DISCUSSION

Our findings suggest that upwelling‐mediated ecological controls (i.e., herbivory, light availability) and preemption interact with algal morphological and functional differences to create contrasting low rocky intertidal macroalgal community dynamics and composition at Raramai and Twelve Mile Beach.

### 4.1 Geophysical differences may underpin the variations in algal community responses

Geophysical differences between coasts on the South Island of New Zealand set the stage for the interpretation of our results. Sites on the east coast of the south island, including Raramai, experience little upwelling, lower nutrients, lower phytoplankton levels, and low colonization rates of sessile invertebrates (Menge et al., 1999, 2003; Menge & Menge, 2013). In contrast, sites on the west coast including Twelve Mile Beach experience intermittent upwelling, higher nutrients, higher phytoplankton levels, and high colonization rates of sessile invertebrates. In this context, we found that herbivory controlled the dynamics and composition of macroalgal communities on the east coast. The diversity of macroalgal functional groups was low at Raramai and was mostly dominated by green algae. Even though grazing effects were stronger at Raramai, herbivores did not shift the algal community composition at this site toward apparently well‐defended and unpalatable algae like those seen Twelve Mile Beach. Potential explanations include: (1) a large brown canopy‐forming species (e.g., *Durvillaea willana*) was more abundant in the low intertidal zone at Raramai and can exert considerable whiplash effects that might limit survival of nearby taxa (Santelices et al., 1980; Schiel, 2004). However, our experiments were sited to avoid proximity to *D. willana* so this possibility seems unlikely. (2) Because the east coast experiences persistent downwelling, low nutrient levels may limit diversification of intertidal algal species (e.g., Bracken & Nielsen, 2004), excluding those species with higher nutritional requirements. (3) Prevalence of green algae could inhibit recruitment of other algal species. For example, Sousa (1979) found that the early successional alga *Ulva*, inhibited perennial red algal recruitment on the southern California coast (Sousa, 1979). Lubchenco and Menge (1978), working in a similar community on the Atlantic coast of New England, experimentally demonstrated that a mat of *Enteromorpha* inhibited colonization of red and brown algae. So early colonists, such as green algae at Raramai, could secure most of the available space/light and resist the colonization of subsequent colonists or suppress the growth of those present by settling on and overgrowing established algae. The inhibition of subsequent recruitment by early species would tend to truncate successional sequences at an early stage (Sousa, 1979), possibly contributing to the high density of green algae in Raramai.

At Twelve Mile Beach, competition (mediated by light availability, herbivory, and nutrients) appeared to be important in controlling the macroalgal communities. Here, intermittent upwelling brings dissolved inorganic nitrogen, a necessity for macroalgae, to the surface and subsequently fuels algal productivity (Bustamante, Branch, & Eekhout, 1995; Bustamante, Branch, Eekhout, Robertson, et al., 1995; Menge et al., 1999). Upwelling thus could generate a more complex community composed of diverse species (e.g., Bracken & Nielsen, 2004). Because of stimulation by bottom‐up effects of nutrients and resulting higher macroalgal growth, space is likely to become limiting and competition can become more intense (Bokn et al., 2003; Dayton, 1971).

Consistent with this scenario, space in Twelve Mile Beach's uncleared plots was limited with bare rock cover being consistently less than 10% throughout the experiment, while bare rock cover in Raramai uncleared plots ranged from 5% to 40%. Additionally, in our experiment, average light levels in unshaded plots during the immersion period were 20% lower at Twelve Mile Beach compared to Raramai. Lower light levels could result from extra shading from the turbid waters of the west coast, potentially due to phytoplankton blooms and sediment suspension (Kavanaugh et al., 2009; Menge et al., 1999, 2003). Thus, fueled by nutrients, limited space, variable light, and weak herbivory, competition could become more important.

Mechanistically, these considerations suggest cascading competitive interactions might begin with herbivores suppressing the abundance of green sheets, providing a scenario favoring well‐defended, unpalatable algae (e.g., coarsely branched and jointed calcareous algae), and a positive response of filamentous algae to light. Hence, we hypothesize that cascading effects of competition resulting from environmental stimuli (i.e., higher nutrients, less space, less light, weak herbivory) likely generated the dynamics and composition of macroalgal communities observed on the west coast.

To our knowledge, few studies have delved into the combined effects of upwelling regime, light availability, herbivory, and preemption on algal community composition. However, our results are consistent with the findings of Nielsen and Navarrete (2004). They found that increased nutrients were strongly associated with positive effects on growth of herbivore‐resistant corticated algae (i.e., coarsely branched algae in our study) but with negative effects on ephemeral algae due to herbivory (Nielsen & Navarrete, 2004). As a result, corticated algal abundance was higher at sites of high upwelling intensity, and ephemeral algae and herbivore biomass were higher at sites of low upwelling intensity.

In cleared plots, herbivory, light availability, and competition apparently strongly influenced successional patterns. On the east coast, bare space remained plentiful in the presence of herbivores and competition appeared less intense (i.e., we found no correlations among the different functional groups), while herbivory and shading generated strong patterns with sheet algal cover being low in the presence of grazers and crustose algal cover being high in the presence of shading. On the west coast, bare space was almost immediately monopolized by sessile invertebrates, which initially dominated space regardless of the treatments. Algal colonization began toward the middle of the experimental period, presumably because predators (sea stars, whelks) consumed the barnacles and mussels (e.g., Menge et al., 1999).

Unfortunately, we were unable to continue these experiments beyond early successional stages due to logistical and financial constraints. We believe that future studies allowing longer succession time to further delineate interactions among organisms are warranted. However, based on trends observed in the experiments in the 5th and 6th months and long‐term (~20 years) observations of high limpet density and large limpet size (Menge et al., 2023), we suggest that in uncleared plots at Raramai, grazers would have kept macroalgae sparse in *+herbivore* treatments, while sheet algae would likely persist in *herbivore* treatments, although winter storms likely would reduce their cover (Figure 6). Final trends in cleared Raramai plots suggested similar long‐term grazing outcomes. A strong effect of light, or in other words desiccation stress, would likely have been suppressing the abundance of crustose algae in both cleared and uncleared plots (Figure 6). Thus, at Raramai under reference conditions, grazers (mostly large limpets) would likely maintain a system with much bare space and little macroalgae for some intermediate successional period until slowly colonizing barnacles and mussels gradually became relatively abundant (Menge et al., 2003, 2023).

Trends in uncleared plots at Twelve Mile Beach are consistent with a longer‐term interpretation of relatively weak effects of grazing and persistence of high abundance of multiple groups of macroalgae which compete for space and preempt sessile invertebrates from colonizing (Figure 7). Changes in cleared plots at Twelve Mile Beach are consistent with earlier experimental results (Menge et al., 1999, 2003). That is, bare space is typically densely colonized by sessile invertebrates in spring (~October/November), which are usually consumed by sea stars within a few months, once again freeing up space (Figure 7). Grazers help keep early colonist abundances low until the next wave of sessile invertebrate recruitment temporarily overwhelms them. Colonization of ephemeral algae and invertebrates, and predation/grazing likely cycle a few times before herbivore‐resistant algae become dominant and preempt the early colonizers.

### 4.2 Study sites may represent coastal geophysical differences

Since we were unable to replicate our study at the site‐within‐region scale, we are limited in our inferences with respect to geophysical modulation of regional effects of herbivory, light availability, and preemption on algal populations. However, long‐term monitoring has shown that both physically and biologically, sites within each of these regions are similar, and different between regions (Menge et al., 2003, 2023, Menge & Menge, 2013; see Figure 3). That is, Twelve Mile Beach has similar dynamics and abundance patterns to a west coast site (Woodpecker Bay) and Raramai has comparable similarities to an east coast site (Box Thumb). Thus, even though we were not able to test regional effects in this study, our findings, along with previous studies, highlight the potential importance of regional geophysical differences in modulating the effects of grazing, light availability, predation, preemptive competition and interference competition, and colonization rates on rocky intertidal algal community structure.

### 4.3 Herbivory

Interactive effects were important in these experiments. For example, grazing appeared to have the strongest effect at Raramai on the east coast, particularly on sheet algae, with the greatest effect occurring in cleared treatments. This result is consistent with the study by Menge et al. (2023) in which macrophyte–herbivore interactions weakened with increasing nutrient inputs. In low‐nutrient environments, strong herbivory and low macrophyte productivity occurred and vice versa in high‐nutrient environments. Specifically, in their 3‐year‐long experiment on the east and west coasts of New Zealand (including Raramai and Twelve Mile Beach), they demonstrated an inverse macrophyte‐herbivore pattern, with herbivory being the strongest in the downwelling sites on the east coast.

The high grazing activity was also related to the abundance of green algae. Molluscan grazers have been documented to preferentially graze small, tender algae with limited structural or chemical means to deter herbivores (e.g., *Ulva*, *Enteromorpha*, *Ceramium*, and *Porphyra*) (Lubchenco, 1978). Green algae are usually a preferred food for molluscan grazers due to its greater palatability (Aguilera & Navarrete, 2007; Crowe et al., 2011).

Across all treatments, Raramai had almost three times as much green sheet and twelve times as much green filamentous algae than Twelve Mile Beach (Figure A2). High abundance of green algae at Raramai may be related to the massive 2016 earthquake that hit the Kaikōura region near where the study site is located. The quake caused a multifault rupture that dramatically altered the geology of the region, causing coastal uplift of up to 6 m along the coast north of Kaikōura, about 20+ km to the north of Raramai (Orchard et al., 2021). Following the disturbance, there was a large‐scale intertidal bloom of ephemeral algae, such as *Ulva*, northward of Kaikōura (Alestra et al., 2021). The bloom seems likely to have resulted from a massive die‐off of molluscan grazers in the region due to the uplift (Schiel et al., 2019). However, our observations at Raramai suggested that the uplift was much smaller, <0.5 m, which likely was still enough to influence green algae colonization.

Why were green algae so sparse at Twelve Mile Beach? Our experiments showed that this sparsity was not because green sheets did not recruit on the west coast; they were capable of colonizing space in cleared plots at Twelve Mile Beach (Figure 8). However, our results show that after colonization they are quickly grazed down by herbivores (Figure 8e,f) and competitively dominated by brown and red sheets in the presence of light (Figure 8g). These results suggest that herbivory may generate strong selection for potentially well‐defended, unpalatable algae on the west coast.

### 4.4 Light availability

Light availability appeared to have strong direct and indirect effects on the abundance and dynamics of these communities. In Twelve Mile Beach uncleared plots, filamentous algae generally proliferated when they were unshaded. In contrast, shading appeared to positively affect calcareous algae, having a strong interaction with crustose and a weak one with jointed calcareous on the east coast. This is consistent with other studies that found shading by canopy‐forming algae and epiphytes can provide calcareous algae protection from harmful effects of light, temperature, or desiccation (Edyvean & Ford, 1984b; Littler, 1972; Steneck & Paine, 1986). These and other studies found that crustose corallines can become bleached (i.e., light damaged) when exposed outside the canopy algae and without an appropriate cover of epiphytes (Edyvean & Ford, 1984a; Figueiredo et al., 2000; Hawkins & Harkin, 1985; Littler, 1973). Curiously, despite our east coast results and these documented effects of shading on calcareous algae, they apparently were unaffected by light exposure on the west coast.

### 4.5 Interspecific competition

We are aware that experiments are required to rigorously test the hypothesis of competition among the algal groups, and follow‐up tests are warranted. Although these were not feasible in the context of this study, the negative correlations among several functional groups in the uncleared plots suggested that interspecific competition likely had a strong effect on the west coast (Table 7), with these effects likely mediated by light. For example, when filamentous algal abundance increased in unshaded uncleared plots, abundance of coarsely branched algae decreased (Figure 7a,c). However, in shaded uncleared plots, where filamentous algal cover remained low, the abundance of coarsely branched was high and persistent (Figure 7b,d). Similarly, in shaded plots, jointed calcareous algal cover was negatively correlated with coarsely branched algal abundance. Further, despite the weak positive correlation with filamentous algae, jointed calcareous algae declined when filamentous cover was high in unshaded plots and varied similarly as filamentous algae in shaded plots. Additionally, jointed calcareous algae was negatively correlated with crustose algae. In this case, the upright turf morphology of the non‐crustose algae was short, usually <2 cm tall, so potential obscuring of the crusts by the turf‐forming algae was unlikely to compromise these correlations. Hence, because of the potentially intense competition among macroalgal functional groups, competitive effects might supersede the effect of light availability for calcareous algae, resulting in their lack of response to shading.

### 4.6 Sessile invertebrates

Consumers can regulate the trajectory of succession by operating as filters during the early stages (Navarrete, 1996; Sousa, 1979). In our experiment, sessile invertebrates tended to be abundant in cleared plots and virtually nonexistent in uncleared plots at Twelve Mile Beach. This suggests strong preemptive competition, with algae inhibiting settlement of mussels and barnacles. Herbivores are known to graze on algal sporelings, diatoms and ephemeral algae, thus temporarily enhancing the recruitment of sessile invertebrates on newly cleared surfaces (Lubchenco & Menge, 1978; Sousa, 1979). However, predators (i.e., sea stars and whelks) may consume these invertebrates and make way for later successional algal species to establish and preempt them (Navarrete, 1996; Wootton, 2002). Although the greater final abundance of sessile invertebrates in unshaded cleared plots without herbivory than in shaded cleared plots without herbivory is interesting, the mechanisms underlying this trend are unclear and warrant further investigation.

## 5 CONCLUSIONS

Besides enriching understanding of the dynamics of these macroalgal assemblages, our results help to clarify an earlier result that indicated strong grazing in the low intertidal zone at Twelve Mile Beach (Menge et al., 1999). In that study, experiments were all “successional;” i.e., plots were cleared before applying herbivore exclusion treatments. In our experiment, we included clearance as a treatment and found a similarly strong effect of grazers in cleared plots as in the prior study (Menge et al., 1999). In contrast, experiments in plots with established algae (uncleared) at Twelve Mile Beach demonstrated weak effects of grazers, with competition, both interference and preemptive, being stronger. Here, light exposure was important in determining the outcome of interactions between dominant algal functional groups. By distinguishing between algal functional groups and including different starting conditions in our design, we have provided a richer understanding of the factors involved in determining low intertidal community structure. Twelve Mile Beach results suggest that the mosaic‐like pattern of bare rock intermingled with diverse turf‐forming algae is driven by a complex array of species interactions, including grazing, predation, preemptive competition and interference competition, colonization rates, and how these are modulated by light availability and the oceanic environment. Raramai results contrast with those at Twelve Mile Beach in showing stronger effects of grazing and relatively weak effects of other interactions, low colonization rates of invertebrates, and light effects limited to crustose algae.

Our findings are consistent with some general tenets of ecology in which (1) herbivores enhance species diversity in highly productive environments and reduce species diversity in unproductive environments (Guerry & Menge, 2017; Hillebrand et al., 2007; Proulx & Mazumder, 1998). (2) Light availability (or shading) is a multifaceted phenomenon that is not merely a lack of light, but rather it takes into account a suite of factors, including low light, together with altered atmospheric and substrate conditions, and biotic interactions (Valladares et al., 2016; Valladares & Niinemets, 2008). And (3) community succession provides a crucial temporal framework in understanding ecological processes and assessing temporal consequences of species interacting with abiotic and biotic factors (Navarrete, 1996; Prach & Walker, 2011; Walker & del Moral, 2008; Wootton, 2002).

## AUTHOR CONTRIBUTIONS

**Barbara J. Spiecker:** Conceptualization (lead); data curation (lead); formal analysis (lead); funding acquisition (supporting); investigation (lead); methodology (lead); project administration (lead); resources (equal); software (supporting); supervision (lead); validation (lead); visualization (lead); writing – original draft (lead); writing – review and editing (lead). **Bruce A. Menge:** Conceptualization (supporting); formal analysis (supporting); funding acquisition (lead); investigation (supporting); methodology (supporting); resources (equal); software (lead); writing – original draft (supporting); writing – review and editing (supporting).

## FUNDING INFORMATION

Funding for this study was provided by Oregon Shell Club and Phycological Society of America Grants‐in‐Aid to BJS and grants from NSF (OCE1735911, DEB 1050694, and DEB 1554702), the David and Lucile Packard Foundation, and the Wayne and Gladys Valley Foundation (BAM).

## Supporting information


Appendix S1

## Data Availability

The data is archived in figshare (10.6084/m9.figshare.24261034).

## References

[ece310704-bib-0001] Aguilera, M. A. , & Navarrete, S. A. (2007). Effects of *Chiton granosus* (Frembly, 1827) and other molluscan grazers on algal succession in wave exposed mid‐intertidal rocky shores of Central Chile. Journal of Experimental Marine Biology and Ecology, 349, 84–98.

[ece310704-bib-0002] Alestra, T. , Gerrity, S. , Dunmore, R. A. , Crossett, D. , Orchard, S. , & Schiel, D. R. (2021). Rocky reef impacts of the 2016 Kaikoura earthquake: Extended monitoring of nearshore habitats and communities to 3.5 years. 253. Fisheries New Zealand.

[ece310704-bib-0003] Bertness, M. D. , Leonard, G. H. , Levine, J. M. , & Bruno, J. F. (1999). Climate‐driven interactions among rocky intertidal organisms caught between a rock and a hot place. Oceologia, 120(3), 446–450. 10.1007/s004420050877 28308021

[ece310704-bib-0004] Bokn, T. L. , Duarte, C. M. , Pedersen, M. F. , Marbà, N. , Moy, F. E. , Barrón, C. , Bjerkeng, B. , Borum, J. , Christie, H. , Engelbert, S. , Fotel, F. L. , Hoell, E. , Karez, R. , Kersting, K. , Kraufvelin, P. , Lindblad, C. , Olsen, M. , Sanderud, K. , Sommer, U. , & Sørensen, K. (2003). The response of experimental rocky shore communities to nutrient additions. Ecosystems, 6(6), 577–594.

[ece310704-bib-0005] Bosman, A. L. , Hockey, P. A. R. , & Siegfried, W. R. (1987). The influence of coastal upwelling on the functional structure of rocky intertidal communities. Oecologia, 72, 226–232. 28311545 10.1007/BF00379273

[ece310704-bib-0006] Bracken, M. E. , Menge, B. A. , Foley, M. M. , Sorte, C. J. , Lubchenco, J. , & Schiel, D. R. (2012). Mussel selectivity for high‐quality food drives carbon inputs into open‐coast intertidal ecosystems. Marine Ecology Progress Series, 459, 53–62.

[ece310704-bib-0007] Bracken, M. E. S. , & Nielsen, K. J. (2004). Diversity of intertidal macroalgae increases with nitrogen loading by invertebrates. Ecology, 85, 2828–2836.

[ece310704-bib-0008] Burnaford, J. L. (2004). Habitat modification and refuge from sublethal stress drive a marine plant–herbivore association. Ecology, 85(10), 2837–2849.

[ece310704-bib-0009] Bustamante, R. H. , Branch, G. M. , & Eekhout, S. (1995). Maintenance of an exceptional intertidal grazer biomass in South Africa: Subsidy by subtidal kelps. Ecology, 76, 2314–2329.

[ece310704-bib-0010] Bustamante, R. H. , Branch, G. M. , Eekhout, S. , Robertson, B. , Zoutendyk, P. , Schleyer, M. , Dye, A. , Hanekom, N. , Keats, D. , Jurd, M. , & McQuaid, C. (1995). Gradients of intertidal primary productivity around the coast of South Africa and their relationships with consumer biomass. Oecologia, 102, 189–201. 28306874 10.1007/BF00333251

[ece310704-bib-0012] Connell, J. H. (1972). Community interactions on marine rocky intertidal shores. Annual Review of Ecology and Systematics, 3, 169–192.

[ece310704-bib-0014] Creese, R. G. (1982). Distribution and abundance of the acmaeid limpet, *Patelloida latistrigata*, and its interaction with barnacles. Oecologia, 52, 85–96. 28310112 10.1007/BF00349015

[ece310704-bib-0015] Crowe, T. P. , Frost, N. J. , & Hawkins, S. J. (2011). Interactive effects of losing key grazers and ecosystem engineers vary with environmental context. Marine Ecology Progress Series, 430, 223–234.

[ece310704-bib-0016] Cubit, J. D. (1984). Herbivory and the seasonal abundance of algae on a high intertidal rocky shore. Ecology, 65(6), 1904–1917.

[ece310704-bib-0017] Dayton, P. K. (1971). Competition, disturbance, and community organization: The provision and subsequent utilization of space in a rocky intertidal community. Ecological Monographs, 41(4), 351–389.

[ece310704-bib-0019] Dunmore, R. A. , & Schiel, D. R. (2003). Demography, competitive interactions and grazing effects of intertidal limpets in southern New Zealand. Journal of Experimental Marine Biology and Ecology, 288, 17–38.

[ece310704-bib-0020] Edyvean, R. G. J. , & Ford, H. (1984a). Population biology of the red crustose alga *Lithophyllum incrustans* Phil. 2. A comparison of populations from three areas of Britain. Biological Journal of the Linnean Society, 23, 353–363.

[ece310704-bib-0021] Edyvean, R. G. J. , & Ford, H. (1984b). Population biology of the crustose red alga *Lithophyllum incrustans*. 3. The effects of local environmental variables. Biological Journal of the Linnean Society, 23, 365–374.

[ece310704-bib-0022] Eriksson, B. K. , Rubach, A. , & Hillebrand, H. (2006). Biotic habitat complexity controls species diversity and nutrient effects on net biomass production. Ecology, 87(1), 246–254. 16634315 10.1890/05-0090

[ece310704-bib-0023] Farrell, T. M. (1988). Community stability: Effects of limpet removal and reintroduction in a rocky intertidal community. Oecologia, 75, 190–197. 28310833 10.1007/BF00378596

[ece310704-bib-0024] Feise, R. J. (2002). Do multiple outcome measures require p‐value adjustment? BMC Medical Research Methodology, 2(1), 8. 12069695 10.1186/1471-2288-2-8 PMC117123

[ece310704-bib-0025] Figueiredo, M. A. , Kain, M. , & Norton, T. A. (2000). Responses of crustose corallines to epiphyte and canopy cover. Journal of Phycology, 36, 17–24.

[ece310704-bib-0026] Freidenburg, T. L. , Menge, B. A. , Halpin, P. M. , Webster, M. , & Sutton‐Grier, A. (2007). Cross‐scale variation in top‐down and bottom‐up control of algal abundance. Journal of Experimental Marine Biology and Ecology, 347(1–2), 8–29.

[ece310704-bib-0027] Gruner, D. S. , Smith, J. E. , Seabloom, E. W. , Sandin, S. A. , Ngai, J. T. , Hillebrand, H. , Haropole, W. S. , Elser, J. J. , Cleland, E. E. , Bracken, M. E. S. , Borer, E. T. , & Bolker, B. M. (2008). A cross‐system synthesis of consumer and nutrient resource control on producer biomass. Ecology Letters, 11(7), 740–755. 18445030 10.1111/j.1461-0248.2008.01192.x

[ece310704-bib-0028] Guerry, A. D. , & Menge, B. A. (2017). Grazer impacts on algal community structure vary with the coastal upwelling regime. Journal of Experimental Marine Biology and Ecology, 488, 10–23.

[ece310704-bib-0029] Guerry, A. D. , Menge, B. A. , & Dunmore, R. A. (2009). Effects of consumers and enrichment on abundance and diversity of benthic algae in a rocky intertidal community. Journal of Experimental Marine Biology and Ecology, 369, 155–164.

[ece310704-bib-0030] Hacker, S. D. , Menge, B. A. , Nielsen, K. J. , Chan, F. , & Gouhier, T. C. (2019). Regional processes are stronger determinants of rocky intertidal community dynamics than local biotic interactions. Ecology, 100(8), e02763. 31127616 10.1002/ecy.2763

[ece310704-bib-0031] Hawkins, S. J. , & Harkin, E. (1985). Preliminary canopy removal experiments in algal dominated communities low on the shore and in the shallow subtidal on the Isle of Man. Botanica Marina, 28, 223–230.

[ece310704-bib-0032] Hay, M. E. (1981). The functional morphology of turf forming seaweeds: Persistence in stressful marine habitats. Ecology, 62, 739–750.

[ece310704-bib-0034] Hillebrand, H. , Gruner, D. S. , Borer, E. T. , Bracken, M. E. , Cleland, E. E. , Elser, J. J. , Harpole, W. S. , Ngai, J. T. , Seabloom, E. W. , Shurin, J. B. , & Smith, J. E. (2007). Consumer versus resource control of producer diversity depends on ecosystem type and producer community structure. Proceedings of the National Academy of Sciences, 104(26), 10904–10909. 10.1073/pnas.0701918104 PMC1904146 17581875

[ece310704-bib-0038] Kavanaugh, M. T. , Nielsen, K. J. , Chan, F. T. , Menge, B. A. , Letelier, R. M. , & Goodrich, L. M. (2009). Experimental assessment of the effects of shade on an intertidal kelp: Do phytoplankton blooms inhibit growth of open coast macroalgae? Limnology and Oceanography, 54, 276–288.

[ece310704-bib-0041] Littler, M. M. (1972). The crustose Corallinaceae. Oceanography and Marine Biology: An Annual Review, 10, 311–347.

[ece310704-bib-0042] Littler, M. M. (1973). The population and community structure of Hawaiian fringing‐reef crustose Corallinaceae (Rhodophyta, Cryptonemiales). Journal of Experimental Marine Biology and Ecology, 11, 103–120.

[ece310704-bib-0044] Littler, M. M. , & Littler, D. S. (1980). The evolution of thallus form and survival strategies in benthic marine macroalgae: Field and laboratory tests of a functional form model. The American Naturalist, 116, 25–44.

[ece310704-bib-0045] Littler, M. M. , & Littler, D. S. (1983). Heteromorphic life‐history strategies in the brown alga *Scytosiphon lomentaria* (Lyngb.) Link. Journal of Phycology, 19(4), 425–431.

[ece310704-bib-0046] Littler, M. M. , & Littler, D. S. (1984). Relationships between macroalgal functional form groups and substrata stability in a subtropical rocky‐intertidal system. Journal of Experimental Marine Biology and Ecology, 74(1), 13–34.

[ece310704-bib-0048] Lubchenco, J. (1978). Plant species diversity in a marine intertidal community: Importance of herbivore food preference and algal competitive abilities. The American Naturalist, 112, 23–39.

[ece310704-bib-0049] Lubchenco, J. (1986). Relative importance of competition and predation: Early colonization by seaweeds in New England. In J. Diamond & T. J. Case (Eds.), Community ecology (pp. 537–555). Harper & Row.

[ece310704-bib-0050] Lubchenco, J. , & Cubit, J. (1980). Heteromorphic life histories of certain marine algae as adaptations to variations in herbivory. Ecology, 61, 676–687.

[ece310704-bib-0051] Lubchenco, J. , & Gaines, S. D. (1981). A unified approach to marine plant‐herbivore interactions. I. Populations and communities. Annual Review of Ecology and Systematics, 12, 405–437.

[ece310704-bib-0052] Lubchenco, J. , & Menge, B. A. (1978). Community development and persistence in a low rocky intertidal zone. Ecological Monographs, 48, 67–94.

[ece310704-bib-0053] Menge, B. A. (1992). Community regulation: Under what conditions are bottom‐up factors important on rocky shores? Ecology, 73(3), 755–765.

[ece310704-bib-0054] Menge, B. A. (2000). Top‐down and bottom‐up community regulation in marine rocky intertidal habitats. Journal of Experimental Marine Biology and Ecology, 250, 257–289. 10969172 10.1016/s0022-0981(00)00200-8

[ece310704-bib-0056] Menge, B. A. , Close, S. L. , Hacker, S. D. , Nielsen, K. J. , & Chan, F. (2021). Biogeography of macrophyte productivity: Effects of oceanic and climatic regimes across spatiotemporal scales. Limnology and Oceanography, 66, 711–726.

[ece310704-bib-0057] Menge, B. A. , Daley, B. A. , Lubchenco, J. , Sanford, E. , Dahlhoff, E. , Halpin, P. M. , Hudson, G. , & Burnaford, J. L. (1999). Top‐down and bottom‐up regulation of New Zealand rocky intertidal communities. Ecological Monographs, 69, 297–330.

[ece310704-bib-0058] Menge, B. A. , Gouhier, T. C. , Hacker, S. D. , Chan, F. , & Nielsen, K. J. (2015). Are meta‐ecosystems organized hierarchically? A model and test in rocky intertidal habitats. Ecological Monographs, 85, 213–233.

[ece310704-bib-0059] Menge, B. A. , Gravem, S. A. , Richmond, E. , & Noble, M. M. (2023). A unified meta‐ecosystem dynamics model: Integrating herbivore‐plant subwebs with the intermittent upwelling hypothesis. Ecosphere, 14(5), e4531.

[ece310704-bib-0060] Menge, B. A. , Lubchenco, J. , Bracken, M. E. S. , Chan, F. , Foley, M. M. , Freidenburg, T. L. , Gaines, S. D. , Hudson, G. , Krenz, C. , Leslie, H. , Menge, D. N. L. , Russell, R. , & Webster, M. S. (2003). Coastal oceanography sets the pace of rocky intertidal community dynamics. Proceedings of the National Academy of Sciences, 100, 12229–12234. 10.1073/pnas.1534875100 PMC218741 14512513

[ece310704-bib-0061] Menge, B. A. , & Menge, D. N. L. (2013). Dynamics of coastal meta‐ecosystems: The intermittent upwelling hypothesis and a test in rocky intertidal regions. Ecological Monographs, 83(3), 283–310.

[ece310704-bib-0063] Navarrete, S. A. (1996). Variable predation: Effects of whelks on a mid‐intertidal successional community. Ecological Monographs, 66(3), 301–321.

[ece310704-bib-0064] Nelson, W. A. (2020). New Zealand seaweeds: An illustrated guide. Te Papa Press.

[ece310704-bib-0065] Nielsen, K. J. (2001). Bottom‐up and top‐down forces in tidepools: Test of a food chain model in an intertidal community. Ecological Monographs, 71, 187–217.

[ece310704-bib-0066] Nielsen, K. J. , & Navarrete, S. A. (2004). Mesoscale regulation comes from the bottom‐up: Intertidal interactions between consumers and upwelling. Ecology Letters, 7(1), 31–41.

[ece310704-bib-0067] Noël, L. M. L. , Griffin, J. N. , Moschella, P. S. , Jenkins, S. R. , Thompson, R. C. , & Hawkins, S. J. (2009). Changfes in diversity and ecosystem functioning during succession. In Marine hard bottom communities: Patterns, dynamics, diversity, and change (pp. 213–223). Springer‐Verlag Berlin Heidelberg.

[ece310704-bib-0068] Orchard, S. , Fischman, H. S. , Gerrity, S. , Alestra, T. , Dunmore, R. , & Schiel, D. R. (2021). Threshold effects of relative sea‐level change in intertidal ecosystems: Empirical evidence from earthquake‐induced uplift on a rocky coast. GeoHazards, 2, 302–320.

[ece310704-bib-0069] Paine, R. T. (1984). Ecological determinism in the competition for space. Ecology, 65, 1339–1348.

[ece310704-bib-0070] Paine, R. T. , & Fenchel, T. (1994). Marine rocky shores and community ecology: An experimentalist's perspective. Vol. 4. Ecology Institute.

[ece310704-bib-0071] Paine, R. T. , & Gould, C. E. (1977). Controlled manipulations in the marine intertidal zone and their contributions to ecological theory. Proceedings of the Academy of Natural Sciences, 12, 245–270.

[ece310704-bib-0072] Perneger, T. V. (1998). What's wrong with Bonferroni adjustments. BMJ, 316(7139), 1236–1238. 9553006 10.1136/bmj.316.7139.1236 PMC1112991

[ece310704-bib-0073] Pfeiffer, T. Ž. , Mihaljević, M. , Špoljarić, D. , Stević, F. , & Plenković‐Moraj, A. (2015). The disturbance‐driven changes of periphytic algal communities in a Danubian floodplain lake. Knowledge and Management of Aquatic Ecosystems, 416, 02.

[ece310704-bib-0074] Prach, K. , & Walker, L. R. (2011). Four opportunities for studies of ecological succession. Trends in Ecology & Evolution, 26(3), 119–123. 21295370 10.1016/j.tree.2010.12.007

[ece310704-bib-0075] Proulx, M. , & Mazumder, A. (1998). Reversal of grazing impact on plant species richness in nutrient‐poor vs. nutrient‐rich ecosystems. Ecology, 79(8), 2581–2592.

[ece310704-bib-0076] Ramus, J. (1978). Seaweed anatomy and photosynthetic performance: The ecological significance of light guides, heterogeneous absorption and multiple scatter. Journal of Phycology, 14, 352–362.

[ece310704-bib-0077] Rilov, G. , & Schiel, D. R. (2006a). Seascape‐dependent subtidal‐intertidal trophic linkages. Ecology, 87(3), 731–744. 16602302 10.1890/04-1853

[ece310704-bib-0078] Rilov, G. , & Schiel, D. R. (2006b). Trophic linkages across seascapes: Subtidal predators limit effective mussel recruitment in rocky intertidal communities. Marine Ecology Progress Series, 327, 83–93.

[ece310704-bib-0079] Rilov, G. , & Schiel, D. R. (2011). Community regulation: The relative importance of recruitment and predation intensity of an intertidal community dominant in a seascape context. PLoS One, 6(8), e23958. 21887351 10.1371/journal.pone.0023958 PMC3162600

[ece310704-bib-0080] Rothman, K. J. (1990). No adjustments are needed for multiple comparisons. Epidemiology, 1, 43–46. 2081237

[ece310704-bib-0081] Santelices, B. , Castilla, J. C. , Cancino, J. , & Schmiede, P. (1980). Comparative ecology of *Lessonia nigrescens* and *Durvillaea Antarctica* (Phaeophyta) in Central Chile. Marine Biology, 59(2), 119–132.

[ece310704-bib-0082] Schiel, D. R. (1990). Macroalgal assemblages in New Zealand: Structure, interactions and demography. Hydrobiologia, 192(1), 59–76.

[ece310704-bib-0083] Schiel, D. R. (2004). The structure and replenishment of rocky shore intertidal communities and biogeographic comparisons. Journal of Experimental Marine Biology and Ecology, 300(1–2), 309–342.

[ece310704-bib-0084] Schiel, D. R. (2011). Biogeographic patterns and long‐term changes on New Zealand coastal reefs: Non‐trophic cascades from diffuse and local impacts. Journal of Experimental Marine Biology and Ecology, 400(1–2), 33–51.

[ece310704-bib-0085] Schiel, D. R. , Alestra, T. , Gerrity, S. , Orchard, S. , Dunmore, R. , Pirker, J. , Lilley, S. , Tait, L. , Hickford, M. , & Thomsen, M. (2019). The Kaikōura earthquake in southern New Zealand: Loss of connectivity of marine communities and the necessity of a cross‐ecosystem perspective. Aquatic Conservation: Marine and Freshwater Ecosystems, 29, 1520–1534.

[ece310704-bib-0086] Schiel, D. R. , Lilley, S. A. , South, P. M. , & Coggins, J. H. (2016). Decadal changes in sea surface temperature, wave forces and intertidal structure in New Zealand. Marine Ecology Progress Series, 548, 77–95.

[ece310704-bib-0088] Sellers, A. J. , Leung, B. , & Torchin, M. E. (2020). Global meta‐analysis of how marine upwelling affects herbivory. Global Ecology and Biogeography, 29, 370–383.

[ece310704-bib-0089] Slocum, C. J. (1980). Differential susceptibility to grazers in two phases of an intertidal alga: Advantages of heteromorphic generations. Journal of Experimental Marine Biology and Ecology, 46, 99–110.

[ece310704-bib-0090] Sousa, W. P. (1979). Experimental investigations of disturbance and ecological succession in a rocky intertidal algal community. Ecological Monographs, 49(3), 227–254.

[ece310704-bib-0091] Sousa, W. P. , Schroeter, S. C. , & Gaines, S. D. (1981). Latitudinal variation in intertidal algal community structure: The influence of grazing and vegetative propagation. Oecologia, 48, 297–307. 28309744 10.1007/BF00346486

[ece310704-bib-0092] Spiecker, B. J. , & Menge, B. A. (2022). El Niño and marine heatwaves: Ecological impacts on Oregon rocky intertidal kelp communities at local to regional scales. Ecological Monographs, 92(2), e1504.

[ece310704-bib-0093] Steneck, R. S. , & Paine, R. T. (1986). Ecological and taxonomic studies of shallow‐water encrusting Corallinaceae (Rhodophyta) of the boreal northeastern Pacific. Phycologia, 25, 221–240.

[ece310704-bib-0094] Stevens, C. L. , O'Callaghan, J. M. , Chiswell, S. M. , & Hadfield, M. G. (2021). Physical oceanography of New Zealand/Aotearoa shelf seas—A review. New Zealand Journal of Marine and Freshwater Research, 55, 6–45.

[ece310704-bib-0095] Tapia, F. J. , Navarrete, S. A. , Castillo, M. , Menge, B. A. , Castilla, J. C. , Largier, J. , Wieters, E. A. , Broitman, B. L. , & Barth, J. A. (2009). Thermal indices of upwelling effects on inner‐shelf habitats. Progress in Oceanography, 83(1–4), 278–287.

[ece310704-bib-0096] Trygonis, V. , & Sini, M. (2012). photoQuad: A dedicated seabed image processing software, and a comparative error analysis of four photoquadrat methods. Journal of Experimental Marine Biology and Ecology, 424‐425, 99–108.

[ece310704-bib-0097] Valladares, F. , Laanisto, L. , Niinemets, Ü. , & Zavala, M. A. (2016). Shedding light on shade: Ecological perspectives of understorey plant life. Plant Ecology and Diversity, 9(3), 237–251.

[ece310704-bib-0098] Valladares, F. , & Niinemets, Ü. (2008). Shade tolerance, a key plant feature of complex nature and consequences. Annual Review of Ecology, Evolution, and Systematics, 39, 237–257.

[ece310704-bib-0099] Ver Hoef, J. M. (2012). Who invented the delta method? The American Statistician, 66, 124–127.

[ece310704-bib-0100] Vermeij, G. J. (1978). Biogeography and adaptation. Harvard University Press p. 332.

[ece310704-bib-0103] Walker, L. R. , & del Moral, R. (2008). Transition dynamics in succession: Implications for rates, trajectories and restoration, Chapter 3. In K. Suding & R. J. Hobbs (Eds.), New Models for Ecosystem Dynamics and Restoration. Island Press.

[ece310704-bib-0104] Warton, D. I. , & Hui, F. K. (2011). The arcsine is asinine: The analysis of proportions in ecology. Ecology, 92(1), 3–10. 21560670 10.1890/10-0340.1

[ece310704-bib-0105] Watt, C. A. , & Scrosati, R. A. (2013). Regional consistency of intertidal elevation as a mediator of seaweed canopy effects on benthic species richness, diversity, and composition. Marine Ecology Progress Series, 491, 91–99.

[ece310704-bib-0106] Wieters, E. A. , Broitman, B. R. , & Brancha, G. M. (2009). Benthic community structure and spatiotemporal thermal regimes in two upwelling ecosystems: Comparisons between South Africa and Chile. Limnology and Oceanography, 54(4), 1060–1072.

[ece310704-bib-0107] Wootton, J. T. (2002). Indirect effects in complex ecosystems: Recent progress and future challenges. Journal of Sea Research, 48(2), 157–172.

