# Supplementary material for: Testing effects of bottom‐up factors, grazing, and competition on New Zealand rocky intertidal algal communities
Source: Ecol Evol. 2024 Mar 7;14(3):e10704. doi: 10.1002/ece3.10704 (PMC10920032; doi:10.1002/ece3.10704)
Supplement: Supplementary file 1 — Appendix S1 [file ECE3-14-e10704-s001.docx]

**Appendix A**

**Authors:** Barbara J. Spiecker and Bruce A. Menge

**Title:** Testing effects of bottom-up factors, grazing and pre-emptive competition in rocky intertidal algal communities on the South Island of New Zealand

**Journal:** Marine Ecology Progress Series

**Table A1. Multiple pairwise comparisons of Site*Treatment*Functional Group for the log(Final Cover + 1) within the cleared plots.** Site abbreviations are: Raramai (RR) and Twelve Mile Beach (TMB). Treatment abbreviations are: +He/-He = presence/absence of herbivores, +Sh/-Sh = shaded/unshaded, -Cl/+Cl = uncleared/cleared.

| **Comparison** | | **Estimate** | **Standard Error** | **DF** | ***t*-value** | ***p*-value** |
| --- | --- | --- | --- | --- | --- | --- |
| RR +He/-Sh/+Cl Sheet | RR -He/-Sh/+Cl Sheet | -1.3294 | 0.3329 | 144 | -3.99 | **0.0001** |
| RR +He/-Sh/+Cl Filamentous | RR -He/-Sh/+Cl Filamentous | -0.7462 | 0.3329 | 144 | -2.24 | **0.0265** |
| RR +He/-Sh/+Cl Crustose | RR -He/-Sh/+Cl Crustose | -0.04402 | 0.3329 | 144 | -0.13 | 0.8950 |
| RR +He/+Sh/+Cl Sheet | RR -He/+Sh/+Cl Sheet | -0.9493 | 0.3329 | 144 | -2.85 | **0.0050** |
| RR +He/+Sh/+Cl Crustose | RR -He/+Sh/+Cl Crustose | -0.06825 | 0.3329 | 144 | -0.21 | 0.8379 |
| RR +He/-Sh/+Cl Sheet | RR +He/+Sh/+Cl Sheet | -0.3705 | 0.3329 | 144 | -1.11 | 0.2676 |
| RR +He/-Sh/+Cl Crustose | RR +He/+Sh/+Cl Crustose | -1.0321 | 0.3329 | 144 | -3.10 | **0.0023** |
| RR -He/-Sh/+Cl Sheet | RR -He/+Sh/+Cl Sheet | 0.009616 | 0.3329 | 144 | 0.03 | 0.9970 |
| RR -He/-Sh/+Cl Filamentous | RR -He/+Sh/+Cl Filamentous | 0.7462 | 0.3329 | 144 | 2.24 | **0.0265** |
| RR -He/-Sh/+Cl Crustose | RR -He/+Sh/+Cl Crustose | -1.0563 | 0.3329 | 144 | -3.17 | **0.0018** |
| TMB +He/-Sh/+Cl Sheet | TMB -He/-Sh/+Cl Sheet | -0.2118 | 0.3329 | 144 | -0.66 | 0.5122 |
| TMB +He/-Sh/+Cl Filamentous | TMB -He/-Sh/+Cl Filamentous | -0.5392 | 0.3329 | 144 | -1.62 | 0.1075 |
| TMB +He/-Sh/+Cl Crustose | TMB -He/-Sh/+Cl Crustose | -0.07138 | 0.3329 | 144 | -0.21 | 0.8305 |
| TMB +He/-Sh/+Cl Invertebrates | TMB -He/-Sh/+Cl Invertebrates | -0.1159 | 0.3329 | 144 | -0.35 | 0.7283 |
| TMB +He/+Sh/+Cl Sheet | TMB -He/+Sh/+Cl Sheet | -0.4934 | 0.3329 | 144 | -1.48 | 0.1405 |
| TMB +He/+Sh/+Cl Filamentous | TMB -He/+Sh/+Cl Filamentous | -1.2422 | 0.3329 | 144 | -3.73 | **0.0003** |
| TMB +He/+Sh/+Cl Crustose | TMB -He/+Sh/+Cl Crustose | -0.1405 | 0.3329 | 144 | -0.42 | 0.6737 |
| TMB +He/+Sh/+Cl Invertebrates | TMB -He/+Sh/+Cl Invertebrates | 0.4502 | 0.3329 | 144 | 1.35 | 0.1784 |
| TMB +He/-Sh/+Cl Sheet | TMB +He/+Sh/+Cl Sheet | 0.6417 | 0.3329 | 144 | 1.93 | 0.0559 |
| TMB +He/-Sh/+Cl Filamentous | TMB +He/+Sh/+Cl Filamentous | 0.1505 | 0.3329 | 144 | 0.45 | 0.6519 |
| TMB +He/-Sh/+Cl Crustose | TMB +He/+Sh/+Cl Crustose | 0.01208 | 0.3329 | 144 | 0.04 | 0.9711 |
| TMB +He/-Sh/+Cl Invertebrates | TMB +He/+Sh/+Cl Invertebrates | 0.2460 | 0.3329 | 144 | 0.74 | 0.4611 |
| TMB -He/-Sh/+Cl Sheet | TMB -He/+Sh/+Cl Sheet | 0.3670 | 0.3329 | 144 | 1.10 | 0.2721 |
| TMB -He/-Sh/+Cl Filamentous | TMB -He/+Sh/+Cl Filamentous | -0.5525 | 0.3329 | 144 | -1.66 | 0.0992 |
| TMB -He/-Sh/+Cl Crustose | TMB -He/+Sh/+Cl Crustose | -0.05703 | 0.3329 | 144 | -0.17 | 0.8642 |
| TMB -He/-Sh/+Cl Invertebrates | TMB -He/+Sh/+Cl Invertebrates | 0.8121 | 0.3329 | 144 | 2.44 | **0.0159** |

**Table A2. Multiple pairwise comparisons of Site*Treatment*Functional Group for log(Final Cover + 1) within uncleared plots.** Site and treatment codes are as in Table A1.

| **Comparison** | | **Estimate** | **Standard Error** | **DF** | ***t*-value** | ***p*-value** |
| --- | --- | --- | --- | --- | --- | --- |
| RR +He/-Sh/-Cl Sheet | RR -He/-Sh/-Cl Sheet | -0.7187 | 0.3756 | 68 | -1.91 | 0.0599 |
| RR +He/-Sh/-Cl Filamentous | RR -He/-Sh/-Cl Filamentous | 0.5267 | 0.5312 | 68 | 0.99 | 0.3250 |
| RR +He/-Sh/-Cl Coarsely Branched | RR -He/-Sh/-Cl Coarsely Branched | 0.2218 | 0.6134 | 68 | 0.36 | 0.7187 |
| RR +He/-Sh/-Cl Jointed Calcareous | RR -He/-Sh/-Cl Jointed Calcareous | 0.08401 | 0.3313 | 68 | 0.25 | 0.8006 |
| RR +He/-Sh/-Cl Crustose | RR -He/-Sh/-Cl Crustose | 0.4103 | 0.3960 | 68 | 1.04 | 0.3038 |
| RR +He/+Sh/-Cl Sheet | RR -He/+Sh/-Cl Sheet | 0.03816 | 0.3313 | 68 | 0.12 | 0.9086 |
| RR +He/+Sh/-Cl Filamentous | RR -He/+Sh/-Cl Filamentous | -0.1105 | 0.5009 | 68 | -0.22 | 0.8260 |
| RR +He/+Sh/-Cl Coarsely Branched | RR -He/+Sh/-Cl Coarsely Branched | 0.4375 | 0.5312 | 68 | 0.82 | 0.4130 |
| RR +He/+Sh/-Cl Jointed Calcareous | RR -He/+Sh/-Cl Jointed Calcareous | 0.4538 | 0.3542 | 68 | 1.28 | 0.2044 |
| RR +He/+Sh/-Cl Crustose | RR -He/+Sh/-Cl Crustose | -0.4302 | 0.3313 | 68 | -1.30 | 0.1985 |
| RR +He/+Sh/-Cl Invertebrates | RR -He/+Sh/-Cl Invertebrates | 0.9031 | 0.6134 | 68 | 1.47 | 0.1456 |
| RR +He/-Sh/-Cl Sheet | RR +He/+Sh/-Cl Sheet | -0.5709 | 0.3960 | 68 | -1.44 | 0.1540 |
| RR +He/+Sh/-Cl Filamentous | RR +He/+Sh/-Cl Filamentous | 0.1181 | 0.6134 | 68 | 0.19 | 0.8479 |
| RR +He/-Sh/-Cl Coarsely Branched | RR +He/+Sh/-Cl Coarsely Branched | -0.03959 | 0.5312 | 68 | -0.07 | 0.9408 |
| RR +He/-Sh/-Cl Jointed Calcareous | RR +He/+Sh/-Cl Jointed Calcareous | -0.6695 | 0.3542 | 68 | -1.89 | 0.0630 |
| RR +He/-Sh/-Cl Crustose | RR +He/+Sh/-Cl Crustose | 0.1421 | 0.3960 | 68 | 0.36 | 0.7208 |
| RR -He/-Sh/-Cl Sheet | RR -He/+Sh/-Cl Sheet | 0.1859 | 0.3067 | 68 | 0.61 | 0.5464 |
| RR -He/-Sh/-Cl Filamentous | RR -He/+Sh/-Cl Filamentous | -0.5191 | 0.3960 | 68 | -1.31 | 0.1943 |
| RR -He/-Sh/-Cl Coarsely Branched | RR -He/+Sh/-Cl Coarsely Branched | 0.1761 | 0.6134 | 68 | 0.29 | 0.7749 |
| RR -He/-Sh/-Cl Jointed Calcareous | RR -He/+Sh/-Cl Jointed Calcareous | -0.2997 | 0.3313 | 68 | -2.11 | **0.0387** |
| RR -He/-Sh/-Cl Crustose | RR-He/+Sh/-Cl Crustose | -0.6984 | 0.3313 | 68 | -2.11 | **0.0387** |
| TMB +He/-Sh/-Cl Filamentous | TMB -He/-Sh/-Cl Filamentous | -0.08638 | 0.3067 | 68 | -0.28 | 0.7791 |
| TMB +He/-Sh/-Cl Coarsely Branched | TMB -He/-Sh/-Cl Coarsely Branched | 0.3065 | 0.3313 | 68 | 0.93 | 0.3582 |
| TMB +He/-Sh/-Cl Jointed Calcareous | TMB -He/-Sh/-Cl Jointed Calcareous | -0.6573 | 0.3313 | 68 | -1.98 | 0.0513 |
| TMB +He/-Sh/-Cl Crustose | TMB -He/-Sh/-Cl Crustose | 0.3365 | 0.3313 | 68 | 1.02 | 0.3133 |
| TMB +He/+Sh/-Cl Filamentous | TMB -He/+Sh/-Cl Filamentous | -0.3263 | 0.5312 | 68 | -0.61 | 0.5412 |
| TMB +He/+Sh/-Cl Coarsely Branched | TMB -He/+Sh/-Cl Coarsely Branched | 0.2157 | 0.3067 | 68 | 0.70 | 0.4843 |
| TMB +He/+Sh/-Cl Jointed Calcareous | TMB -He/+Sh/-Cl Jointed Calcareous | 0.4664 | 0.3313 | 68 | -0.97 | 0.3350 |
| TMB +He/+Sh/-Cl Crustose | TMB -He/+Sh/-Cl Crustose | -0.3217 | 0.3313 | 68 | -0.97 | 0.3350 |
| TMB +He/-Sh/-Cl Filamentous | TMB +He/+Sh/-Cl Filamentous | 0.3148 | 0.4850 | 68 | 0.65 | 0.5184 |
| TMB +He/-Sh/-Cl Coarsely Branched | TMB +He/+Sh/-Cl Coarsely Branched | -0.4876 | 0.3067 | 68 | -1.59 | 0.1165 |
| TMB +He/-Sh/-Cl Jointed Calcareous | TMB +He/+Sh/-Cl Jointed Calcareous | -0.3337 | 0.3313 | 68 | -1.01 | 0.3173 |
| TMB +He/-Sh/-Cl Crustose | TMB +He/+Sh/-Cl Crustose | -0.3737 | 0.3067 | 68 | -1.22 | 0.2273 |
| TMB -He/-Sh/-Cl Filamentous | TMB -He/+Sh/-Cl Filamentous | 0.07497 | 0.3756 | 68 | 0.20 | 0.8424 |
| TMB -He/-Sh/-Cl Coarsely Branched | TMB -He/+Sh/-Cl Coarsely Branched | -0.5784 | 0.3313 | 68 | -1.75 | 0.0853 |
| TMB -He/-Sh/-Cl Jointed Calcareous | TMB -He/+Sh/-Cl Jointed Calcareous | 0.7899 | 0.3313 | 68 | 2.38 | **0.0199** |
| TMB -He/-Sh/-Cl Crustose | TMB -He/+Sh/-Cl Crustose | -1.0319 | 0.3542 | 68 | -2.91 | **0.0048** |

**Figure A1. Herbivory (present/absent), light availability (unshaded/shaded), and pre-emption competition (cleared/uncleared) experiment set up.** Uncleared plots consisted of: (A) -Herbivore -Shade, (B) +Herbivore -Shade, (C) -Herbivore +Shade, and (D) +Herbivore +Shade. Cleared plots had the same set up except the plots were initially cleared. The shade treatment used two layers of black plastic mesh fastened to the rocks, covering macroalgae in a domed fashion. The herbivory treatment used a Z-spar marine epoxy band around exclusion plots and the epoxy band was painted with copper-based antifouling paint. The pre-emption treatment cleared the plots of algae and invertebrates with a chisel and a wire brush and sprayed with oven cleaner to remove algal crusts and diatoms still adhering to the rock.

**Figure A2. Average composition of sheet (A) and filamentous algae (B) (all treatments combined) at Raramai and Twelve Mile Beach.** Percent algal composition was standardized to 100% to acquire proportional cover for each phylum. The site abbreviations are: RR = Raramai and TMB = Twelve Mile Beach. Algal phyla are color coded as Green = green fill and Brown/red = orange fill.
